# Pooled extracellular receptor-ligand interaction screening using CRISPR activation

**DOI:** 10.1186/s13059-018-1581-3

**Published:** 2018-11-26

**Authors:** Zheng-Shan Chong, Shuhei Ohnishi, Kosuke Yusa, Gavin J. Wright

**Affiliations:** 1 Cell Surface Signalling Laboratory, Wellcome Sanger Institute, Cambridge, CB10 1SA UK; 2 Stem Cell Genetics Laboratory, Wellcome Sanger Institute, Cambridge, CB10 1SA UK

**Keywords:** Cell surface receptors, Cell signaling, CRISPR activation, Extracellular protein interactions, Flow cytometry, Genome-wide screening, G-protein-coupled receptor, Monoclonal antibodies

## Abstract

Extracellular interactions between cell surface receptors are necessary for signaling and adhesion but identifying them remains technically challenging. We describe a cell-based genome-wide approach employing CRISPR activation to identify receptors for a defined ligand. We show receptors for high-affinity antibodies and low-affinity ligands can be unambiguously identified when used in pools or as individual binding probes. We apply this technique to identify ligands for the adhesion G-protein-coupled receptors and show that the Nogo myelin-associated inhibitory proteins are ligands for ADGRB1. This method will enable extracellular receptor-ligand identification on a genome-wide scale.

**Electronic supplementary material:**

The online version of this article (10.1186/s13059-018-1581-3) contains supplementary material, which is available to authorized users.

## Background

Identifying cell surface receptors for ligands such as proteins, small molecules, or whole pathogens, is an important step towards understanding how intercellular signaling events are initiated and discovering new drug targets. Because the extracellular regions of receptors are directly accessible to systemically delivered therapeutics, particularly monoclonal antibodies, these proteins and their interactions are highly valued targets and continue to represent a large fraction of currently approved drugs [1]. However, because solubilizing membrane-embedded receptor complexes in a solvent that retains their native conformation is challenging, and their interaction affinities are often very weak, it is difficult to detect this class of protein-protein interaction using most commonly employed methods [2].

One successful approach for large-scale extracellular interaction screening relies on detecting direct interactions within large libraries of soluble recombinant proteins representing the entire extracellular regions of cell surface proteins [3–5]. In such assays, bait proteins are captured in addressed arrays and tested for direct binding with prey proteins that are oligomerized to increase local avidity and permit the detection of even very weak interactions. While this approach has enabled the construction of extracellular protein-protein interaction networks [4–6], creating comprehensive libraries containing thousands of different recombinant proteins is impractical for most laboratories. In addition, this general method is largely limited to cell surface proteins that contain a single contiguous ectodomain which means that receptors that span the membrane multiple times are not accessible by this method—a serious limitation given that they represent over half of all cell surface proteins encoded in the human genome. Together, these constraints make this approach unsuitable for most laboratories who are usually interested in identifying the receptor for one or a few defined ligands rather than interaction networks within larger collections of receptors.

Another successful strategy for receptor identification uses gain of binding function by overexpressing cDNAs encoding cell surface receptors in cells. The identity of the interacting receptor can be determined by an iterative expression cloning approach with a suitable cDNA library [7] or more recently by maintaining large collections of cloned and sequenced cDNA expression plasmids [8–10]. While this approach has the advantage of being able to access different architectural classes of receptor such as those that span the membrane multiple times, creating and maintaining comprehensive cDNA libraries containing thousands of individual clones is very resource-intensive and so this is not a realistic option for most laboratories. Other approaches such as cross-linking chemically derivatized binding probes to receptors to purify and identify them by mass spectrometry look promising once sufficient amounts of receptor-expressing cellular material can be isolated [11, 12]. In summary, there is a pressing need for new systematic approaches to identify receptors for defined ligands that encompasses all receptor architectural classes encoded within the human genome.

The recent development of CRISPR/Cas9-based tools for transcriptional activation of endogenous genes (CRISPRa) has enabled convenient genome-scale cell-based gain-of-function screens to be performed [13, 14]. These methods typically employ a mutant Cas9 protein that lacks nuclease activity to specifically recruit transcriptional activators to promoter regions by complexing it with guide RNAs (gRNAs). Libraries of pooled gRNAs designed to target promoter regions are used to overexpress individual genes from their endogenous loci in mammalian cells irrespective of transcript length and cells displaying the desired phenotype isolated. Importantly, these short (~ 20 nucleotides) gRNAs can serve as molecular barcodes that are easily quantified by modern sequencing technologies to determine which overexpressed gene products are responsible for the desired phenotype. Here, we use CRISPR/Cas9-based transcriptional activation to target all predicted cell surface proteins encoded in the human genome and establish experimental parameters for extracellular interaction screening. We show that this approach can be used to identify receptors for antibodies and endogenous ligands with high statistical confidence and systematically screen for novel extracellular receptor-ligand interactions.

## Results

### CRISPR activation induces rapid overexpression of cell surface receptors

**Fig. 1:**
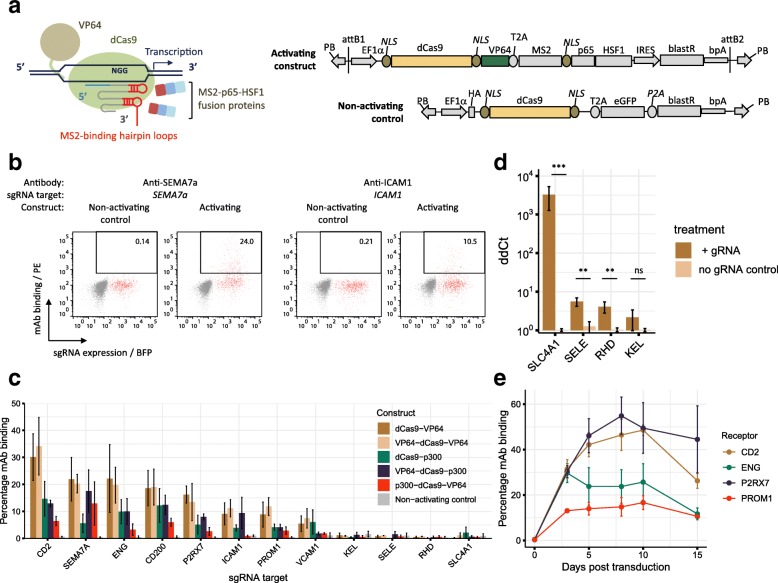
CRISPRa induces upregulation of cell surface protein levels. **a** Schematics of the dCas9 fusion protein and gRNA-dependent recruitment of the VP64 and MS2-p65-HSF1 transcriptional activators to gene promoter regions (left), and transposon-based plasmids for CRISPR-based transcriptional activation and non-activating control (right). **b** Exemplar cytometry plots showing heterogenous upregulation of SEMA7A and ICAM1 at the surface of HEK293 cells 48 h after transduction at low MOI with a lentivirus-delivered pool of eight gRNAs and transfection with either activating (dCas9-VP64) or non-activating control (dCas9) plasmids. gRNA positive cells are represented by red dots, gRNA negative cells in gray. **c** Quantification of cell surface receptor protein upregulation on cells transduced with lentiviruses encoding gRNAs corresponding to the appropriate gene using five different dCas9 activator constructs relative to a non-activating dCas9 control. **d** qRT-PCR analysis of relative mRNA abundance of indicated target genes in cells 48 h post co-transfection with dCas9-VP64 and either targeting gRNA (+) or no gRNA control. Transcript abundance was normalized to *CYPA* expression; bars represent mean ± s.e.m.; *n* = 6. *P* values calculated using a Student’s *t* test, ns *P* > 0.05; **P ≤ 0.01; ***P ≤ 0.001. **e** Percentage of cells expressing the indicated cell surface receptors as determined by mAb staining after transduction of the cloned activator cell line, HEK293-V2M, with appropriate pooled gRNAs. Data points in **c** and **e** represent mean ± s.e.m.; *n* = 3

CRISPRa upregulates the transcription of gRNA-specified genes [13–19]; however, few studies have directly investigated its effects on protein abundance within individual cells and, in the case of cell surface receptors, whether the proteins are displayed on the plasma membrane [20]. Starting with the synergistic activation mediator approach of Konermann et al. [14], which uses both a VP64 transcriptional activator fused to the C-terminus of a deactivated Cas9 (dCas9) protein and recruitment of p65 and HSF1 through an MS2 fusion protein to stem loop structures added to modified gRNAs, we generated a single plasmid that incorporated all these elements (Fig. 1a). To determine if CRISPRa can increase cell surface protein expression, we selected a set of 12 receptors not expressed on HEK293 cells, for which monoclonal antibodies were available. These included receptors restricted to terminally differentiated cell types such as erythrocytes (KEL, RHD, SLC4A1) and T-lymphocytes (CD2). We designed 8 different gRNAs to each of the 12 promoters (Additional file 1: Table S1) and cloned them into a lentiviral plasmid as a pool for each gene (Additional file 2: Figure S1a). HEK293 cells were first transduced at a low multiplicity of infection (MOI) so that each cell typically received only a single gRNA from the pool and then transfected the cells with the activator plasmid before quantifying the level of induced receptor protein overexpression by antibody staining using flow cytometry. Elevated cell surface protein expression was observed within 48 h post-transfection and was highly variable with only a proportion (up to ~ 30%) of the transduced cells exhibiting upregulation of the target receptor and at a wide range of expression levels; this is shown in detail for SEMA7A and ICAM1 (Fig. 1b) and was consistently observed for all tested genes (Fig. 1c). Of the 12 receptors tested, an increase in the cell surface protein expression was observed for eight, including the T-lymphocyte-restricted CD2 receptor (Fig. 1c). By individually testing each of the gRNAs targeting the promoter regions of receptors that could be upregulated, we could show that their efficiency varied significantly: some gRNAs were unable to upregulate protein expression at all; and for those that were, they showed heterogeneity both in the brightness of antibody staining and the proportion of cells stained (Additional file 2: Figure S1b). Three of the four proteins that could not be upregulated were restricted to erythrocytes, suggesting that cell-type specializations such as chromatin structure, which is known to affect the efficiency of CRISPR activation [21], precluded surface expression of these proteins in HEK293 cells. Since directing epigenetic modifiers to enhancer and promoter regions can also induce transcriptional activation [22], we generated a variety of dCas9 fusion constructs containing combinations of the transcriptional activator VP64 and the histone acetyltransferase (HAT) domain of p300, with the aim of improving the efficiency of receptor upregulation (Additional file 2: Figure S1c). We found that the addition of p300 HAT domain did not improve upregulation of erythrocyte-specific proteins (Fig. 1c). In addition, one of these genes, *SLC4A1*, showed more than a 1000-fold increase in mRNA abundance in the presence of dCas9-VP64 and appropriate gRNAs (Fig. 1d), suggesting that the lack of protein expression could be due to a posttranscriptional effect such as cell-type-specific protein trafficking, rather than the lack of transcriptionally permissive chromatin structure. In an attempt to further increase the efficiency of receptor upregulation, we generated a cell line that stably expressed the double VP64-dCas9-VP64 activator construct and selected a clone that showed the highest CRISPRa activity in a reporter assay (Additional file 2: Figure S1d). This efficient activator stable cell line (HEK293-V2M) showed an increase in CRISPRa efficiency compared to transient transfection with dCas9-activators and exhibited sustained overexpression for up to 2 weeks post-transduction (Fig. 1e). These data demonstrate that cell surface receptor proteins can be upregulated and maintained on the surface of cells using CRISPRa.

### Genome-scale enrichment-based extracellular receptor interaction screening by CRISPRa

**Fig. 2:**
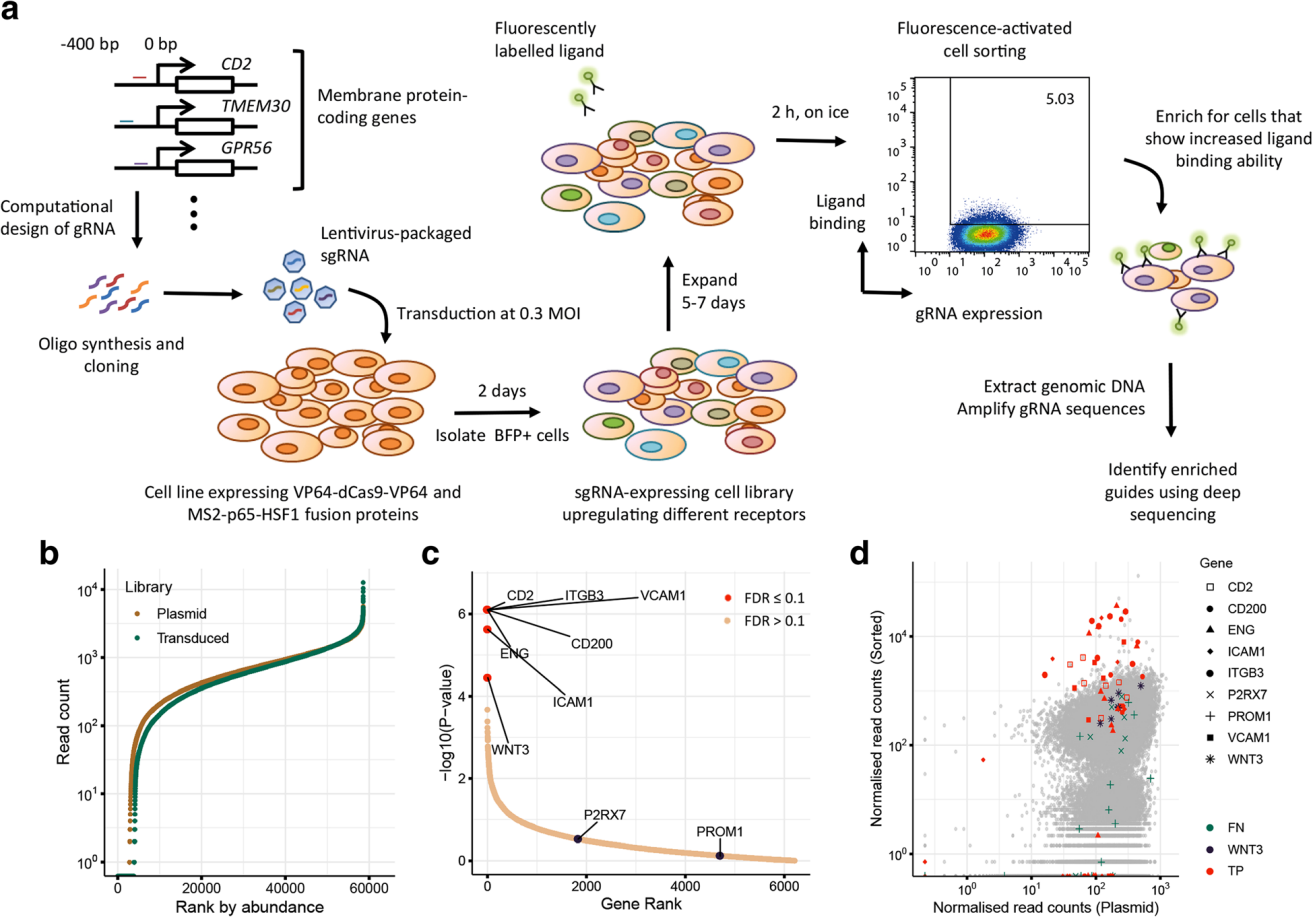
Genome-scale enrichment-based extracellular interaction screen identifies multiple antibody targets. **a** Workflow of CRISPRa screen for identifying extracellular interactions. **b** Ranked gRNA abundance in the plasmid library and cells transduced with the CRISPRa lentiviral library and cultured for 7 days as determined by raw read counts from deep sequencing of PCR-amplified products. **c** Plot of transformed *P* values versus genes in enriched rank order from cells selected using a pool of mAbs targeting eight cell surface proteins: CD2, ITGB3, CD200, VCAM1, ENG, ICAM1, P2RX7, and PROM1. Genes with a false discovery rate (FDR) ≤ 0.1 are indicated with a red dot and labeled. WNT3 was identified as a false positive at this stringency threshold, and P2RX7 and PROM1 as false negatives. **d** Comparison of gRNA sequencing read counts in fluorescence-sorted cells versus the original plasmid library. gRNA targeting the eight genes and WNT3 are denoted with different shapes, gray dots represent gRNA targeting the promoter regions of all other genes. FN, false negative; FP, false positive; TP, true positive

To use CRISPRa for genome-wide receptor screening, we designed a lentiviral library containing gRNAs that targeted the promoter regions of all genes encoding a cell surface protein in the human genome (Fig. 2a). We selected transcripts encoding proteins predicted to contain a transmembrane-spanning region, as well as any other proteins with evidence of association with the plasma membrane using lenient thresholds. Transcription start site (TSS) predictions used were from Gencode v19 TSS stratified by strict Fantom5 CAGE clusters, and if a gene had more than one TSS, the two broadest peaks per gene were selected for increased sensitivity to alternative transcripts [23]. Because of the variation in the ability of individual gRNAs to upregulate cell surface protein levels, we ensured that the majority of promoter regions were targeted by seven different guides (Additional file 2: Figure S2a). The final gRNA library contained 58,071 guides targeting 6213 genes, along with 500 non-targeting control guides [24]. These 6213 genes also include intracellular transmembrane proteins, some of which may be transiently transported to the plasma membrane. Deep sequencing of the plasmid library and cells transduced with the same library after 7 days of culture showed that 89% of guides had read counts within two orders of magnitude demonstrating that library complexity is maintained (Fig. 2b), and this was retained for up to 12 days in culture (Additional file 2: Figure S2b). Individual validation of 34 gRNAs targeting four different genes chosen from the library also demonstrated the success of our design algorithms in selecting guides capable of inducing cell surface receptor protein upregulation and additionally demonstrated that the levels of receptors endogenously expressed by HEK293 cells, such as CD55, can be further increased (Additional file 2: Figure S2c, d).

To establish the experimental parameters necessary for enrichment selections using the genome-wide gRNA library, we iteratively performed a proof-of-principle screen using a pool of monoclonal antibodies recognizing eight different cell surface antigens (Fig. 2a). High CRISPRa activity HEK293-V2M cells were transduced at a low MOI to generate a population of cells each overexpressing a different cell surface receptor and untransduced cells removed by BFP expression-based cell sorting after 48 h. 1 × 10^8^ transduced cells were stained with the pool of eight mAbs and sorted by staining intensity. The relative gRNA abundance within the selected cells and the original plasmid library were quantified by deep sequencing and enrichment analysis performed with MAGeCK [25]. We found that by selecting the brightest 5% of cells and using a false discovery rate (FDR) of < 0.1, we were able to unequivocally identify six out of the eight target antigens with only a single false positive (*WNT3*), which would be expected at this significance threshold (Fig. 2c, Additional file 2: Figure S2e). Accordingly, we observed clear enrichment in individual gRNAs targeting these receptors in sorted cells relative to the plasmid library (Fig. 2d). Two target antigens were not identified (PROM1 and P2RX7) suggesting the guides in our membrane protein library could not sufficiently upregulate these proteins. These observations demonstrate the feasibility of using this approach to unequivocally identify cell surface receptors even within complex pools of ligands.

### Identification of endogenous low-affinity receptor-ligand interactions by CRISPRa

**Fig. 3:**
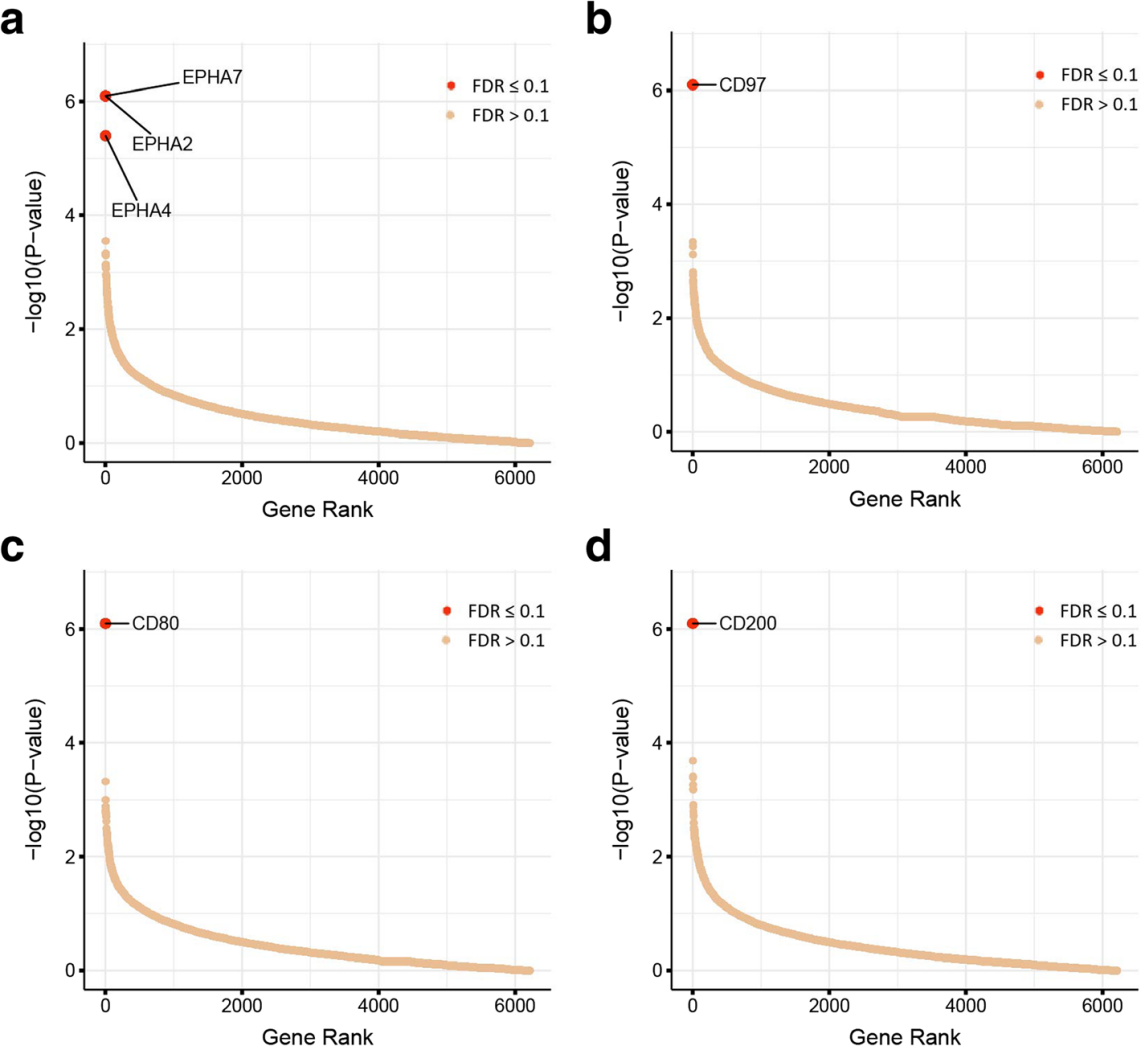
Unequivocal identification of low-affinity endogenous receptor-ligand interactions using CRISPRa. Transformed gene-level enrichment *P* values are plotted against rank-ordered genes for receptor CRISPRa cell selections performed using the ectodomains of EFNA1 (**a**), CD55 (**b**), CTLA4 (**c**), and rat Cd200r (**d**). Screens were conducted in duplicate

While we could show that the CRISPRa enrichment approach successfully identified the binding partners for antibodies, this class of interactions typically has strong interaction affinities that can withstand wash steps thereby facilitating their use in cellular selection assays. By contrast, interactions between endogenous membrane-embedded receptors often have low affinities which makes detecting them technically challenging [26]; for example, the CD97-CD55 interaction is extremely weak (*K*_D_ ~ 86 μM) [27]. To determine if CRISPRa can be used to identify low-affinity extracellular interactions, we expressed a panel of recombinant ectodomains of cell surface proteins with known receptors of low affinity (Additional file 2: Figure S3a). The ectodomains of human EFNA1, CTLA4, CD55, and rat Cd200r were produced as His-tagged, monobiotinylated proteins and clustered around fluorescently labeled streptavidin to increase local binding avidity, and used to select receptor-expressing cells transduced with the membrane protein CRISPRa library. Clear enrichments of gRNA sequences corresponding to expected binding partners for all ligands were observed at a stringent FDR threshold of < 0.1 (Fig. 3a–d and Additional file 2: Figure S3b) with no unexpected receptors demonstrating the low false positive rate of this approach. Notably, the EFNA1 probe bound untransduced HEK293 cells (Additional file 2: Figure S3c) and still identified three Ephrin type-A receptors (EPHA2, 4, and 7) demonstrating that the CRISPRa approach can identify multiple receptors in a single experiment, even though a binding partner is already expressed by the cell line.

### Identification of ligands for adhesion G-protein-coupled receptors

**Fig. 4:**
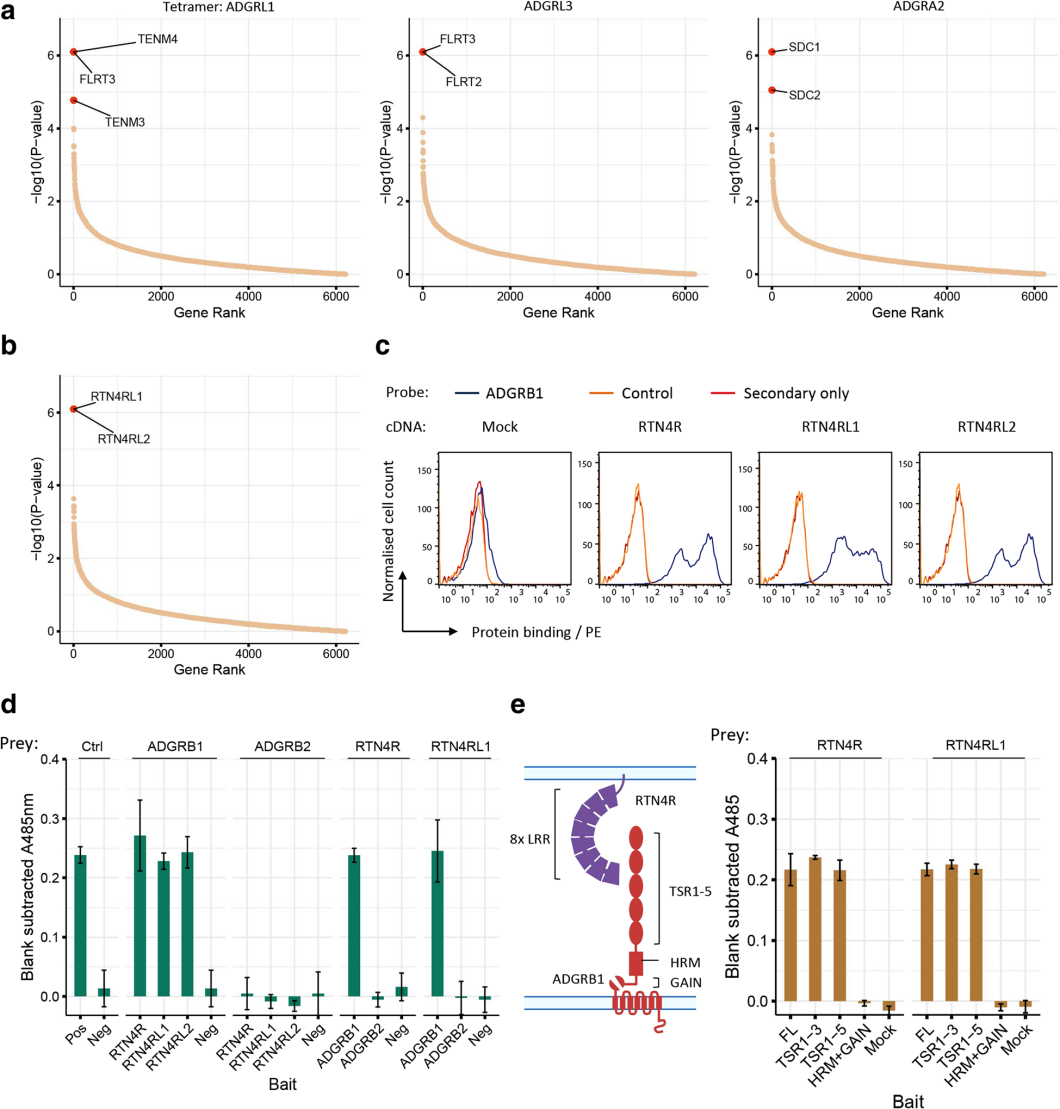
Genome-wide CRISPRa selections identified known and novel ligands for adhesion GPCRs. Transformed gene enrichment *P* values plotted against a rank-ordered gene list for CRISPRa enrichment screens with cells selected using adhesion GPCR recombinant binding probes for ADGRL1, ADGRL3, and ADGRA2 (**a**), and ADGRB1 (**b**). A single screen was performed for each adhesion GPCR bait. **c** A highly-avid fluorescently labeled ADGRB1 binding probe stained cells transfected with cDNAs encoding full-length RTN4R, RTN4RL1, and RTN4RL2 (blue lines) but not mock-transfected cells compared to a control ADGRL1 binding probe (orange line), or streptavidin-phycoerythrin alone (red line). A representative of four independent experiments is shown. **d** The ectodomains of ADGRB1 and RTN4R family members directly interact. The extracellular regions of the named receptors were expressed as soluble biotinylated bait proteins, captured in individual wells of a streptavidin-coated plate and probed for interactions with pentameric beta-lactamase-tagged prey proteins using AVEXIS. Binding is quantified by absorbance at 485 nm of a hydrolysis product of the colorimetric beta-lactamase substrate, nitrocefin. Bars represent blank-subtracted mean ± s.d; *n* = 3. CD97-CD55 interaction was used as a positive control; negative control bait was the CD55 ectodomain. **e** The RTN-family binding interface on ADGRB1 is composed of the N-terminal three TSR domains. Schematic of the RTN-family and ADGRB1 proteins showing their domain organization. Binding of RTN4R and RTN4RL1 preys to fragments of ADGRB1 encompassing the full-length ectodomain (FL), thrombospondin repeats 1–3 (TSR1–3), TSRs 1–5, or the hormone receptor motif and GAIN domain (HRM+GAIN). Bars represent mean ± s.d.; *n* = 3

Adhesion G-protein-coupled receptors (GPCRs) form a large subgroup of the GPCR superfamily, which is a major class of drug targets. Adhesion GPCRs have diverse functions including immune regulation [28, 29], central nervous system development [30], and angiogenesis [31, 32]. These receptors have large extracellular N-terminal regions containing protein domains involved in adhesion [33] and a conserved GPCR proteolysis site (GPS) within an autoproteolysis-inducing domain [34]. These receptors can be activated by ligand binding which relieves the auto-inhibitory action of the receptor ectodomain [35, 36]; crucially, activating ligands for the majority of adhesion GPCRs are not known. To characterize adhesion GPCR ligands, we expressed the entire ectodomains of adhesion GPCRs as soluble recombinant monobiotinylated proteins by mutating the GPS site to prevent proteolysis, made highly avid fluorescent binding probes, and screened by enrichment CRISPRa to identify ligands (Additional file 2: Figure S4a, b). At the stringent significance threshold (FDR < 0.1), we identified binding partners for four receptors which included previously reported interactions between members of the Latrophilin subfamily (ADGRL1 and 3) with FLRT proteins [30] and Tenurins [37] (Fig. 4a). For selections with ADGRA2, we observed an enrichment of guides targeting Syndecans (SDC1 and SDC2), a family of heparan sulfate proteoglycans, which is consistent with known interactions of ADGRA2 with glycosaminoglycans [38] (Fig. 4a). Using ADGRB1 as a selection probe, we observed an enrichment of guides targeting two members of the Reticulon 4 receptor family, RTN4RL1 and 2 (Fig. 4b, Additional file 2: Figure S4c). ADGRB1 (brain angiogenesis inhibitor 1, BAI1) is a phosphatidylserine receptor on professional phagocytes [39] and is enriched in the postsynaptic density in neurons where it regulates excitatory synapse formation in hippocampal and cortical cultures [40] but has no documented ligands in the nervous system. RTN4RL1 and RTN4RL2 (Nogo receptor-like 2 and 3) are both glycosylphosphatidylinositol (GPI)-linked membrane proteins and known to be involved in regulating axon growth and synapse formation, most notably through interactions between RTN4R and the myelin-associated inhibitor, Nogo-66 [41]. To verify these interactions, we individually overexpressed all three members of the Nogo receptor family by transfecting HEK293 cells with cDNA expression plasmids and confirmed gain of cell surface binding with ADGRB1 (Fig. 4c), and demonstrated that this gain of ADGRB1 binding was not due to increased levels of exposed phosphatidylserine on transfected cells (Additional file 2: Figure S4d). To show that the ectodomains of ADGRB1 and RTN4Rs directly interacted, we expressed the extracellular domains of ADGRB1 and RTN4Rs as either soluble recombinant monomeric biotinylated “baits” or pentameric “preys” suitable for avidity-based extracellular interaction screening (AVEXIS) [3]. We observed that ADGRB1, but not the closely related ADGRB2, interacted directly with all three members of the RTN4R family in both bait-prey orientations (Fig. 4d) and showed that the N-terminal thrombospondin type 1 repeats 1–3 on ADGRB1 were sufficient for this binding (Fig. 4e).

## Discussion

We have developed a pooled cell-based screening approach to identify extracellular protein interactions by overexpression of cell surface receptors using CRISPR activation. Existing technologies for large-scale interaction screening such as AVEXIS or cDNA overexpression arrays require creating and maintaining large reagent collections containing thousands of individual plasmids and proteins, representing a significant investment of time and resources. Pooled CRISPRa screening overcomes this difficulty by relying on less costly pooled libraries of short gRNA for endogenous upregulation of surface receptors and requires only one round of selection in a single tube. Additionally, the cell-based aspect of CRISPRa screening provides an advantage over recombinant protein-based assays by allowing the investigation of a wider range of cell surface receptors, for instance those with multiple transmembrane domains or heteromeric receptors. CRISPRa differs from cDNA overexpression through its targeting of endogenous promoters, thereby capturing the variety of isoforms that would be natively transcribed, which could be favorable for agnostic, large-scale screening [10]. The use of a single, easy to transduce activator cell line for CRISPRa screening and the ability to detect multiple receptors regardless of endogenous expression also provides an advantage over CRISPR loss-of-function approaches to identify receptors [42] in situations involving more complex binding profiles, or ligand-binding to specialized cell types, which can be difficult to transduce on the scale required for CRISPR/Cas-9 screening.

We demonstrated the ability of this approach to accurately identify known extracellular protein interactions using both high-affinity mAbs and endogenous receptor-ligand interactions with a low false positive rate. Using a fixed significance threshold for all our experiments, we observed only one unexpected interaction: the identification of *WNT3* from a selection using a pool of antibodies. This gene was identified at a lower threshold than the other targets and may be due to antibody cross-reactivity which is not infrequently observed [43]. In some instances, we were unable to detect enrichment of all expected binding partners including CD86 for CTLA4 and one member of the RTN4R family for ADGRB1. Given that we know the selection probe was active in both these instances, these false negatives are likely to be due to the failure to upregulate these receptors at the cell surface. Based on our observations, this could be due to gRNAs targeting inaccessible promoter regions, alternative or cryptic TSSs, TSS misprediction, or a lack of accessory factors for functional presentation on the surface. An additional possibility is that the receptor may be expressed on the surface, but lacks the necessary components for proper folding and binding to its endogenous ligands. The issues of TSS misprediction and inactive guides are not shared with existing technologies such as cDNA overexpression, highlighting the importance of complementary screening approaches. Nonetheless, this approach should improve with advances in activating gRNA design for CRISPRa, better TSS prediction, and the use of cell lines other than HEK293 cells for screening. Although we did not observe this with the HEK293 cells used here, the expansion of transduced cell libraries may also result in a loss of guides targeting receptors that cause cell toxicity or growth disadvantages when overexpressed in other cell lines.

Using this CRISPRa approach, we identified a novel set of receptor-ligand interactions between ADGRB1 and members of the RTN4R family. The genes encoding all interacting proteins exhibit enriched expression in the brain, and all have documented functions in the regulation of neurite growth and synapse formation both in vitro and in vivo [40, 44]. The RTN4R family of proteins is known to function redundantly with regard to regulating neuronal growth in vivo [44], and therefore, the discovery of common binding partners may provide an explanation for this functional redundancy.

Potential applications for extracellular interaction profiling using CRISPRa are not restricted to proteins such as antibodies or recombinant proteins, but could be used with any selectable probe ranging from small molecules to whole pathogens such as virions or bacteria. Ideally, selection probes would be fluorescently labeled, although this approach should also be compatible with cell viability assays. Because the selection and activation effect are compartmentalized in individual cells, all potential receptors for a given binding probe can, in principle, be identified in a single experiment, even for very complex samples such as polyclonal antibody profiles in serum.

## Conclusions

Identifying extracellular protein interactions between membrane-embedded receptors remains technically challenging because they often have weak interaction affinities, and their amphipathic character makes them difficult to solubilize in their native conformation. Existing methods to detect this class of protein interaction are impractical for most laboratories because they require creating and maintaining thousands of individual plasmids and proteins, lack genome-wide coverage, and are usually restricted to the receptor architectural class that span the membrane a single time. The approach we describe here combines existing strategies of increasing interaction avidity by multimerization with transcriptional upregulation by CRISPRa to provide a method that is cheaper and easier to implement than existing approaches and obviates the need to compile large reagent resources. Since extracellular protein interactions can be targeted by systemically delivered drugs, we envisage that this approach will be very useful to identify novel drug and vaccine targets for genetic and infectious diseases.

## Methods

### Generation of different dCas9 fusion constructs

Expression plasmid pPB-R1R2_EF1adCas9VP64_T2A_MS2p65HSF1-IRESbsdpA was constructed as follows: dCas9VP64_Blast (BsiWI-EcoRI fragment from Addgene 61425) and MS2p65HSF1_hyg (BsiWI-EcoRI fragment from Addgene 61426) were first transferred into pKLV2-EF1a [45], after introducing the BsiWI-EcoRI site between AscI and NotI sites, resulting in pKLV2-EF1adCas9VP64T2ABsd-W and pKLV2-EF1aMS2p65HSF1hyg-W, respectively. To combine dCas9VP64 and MS2p65HSF1 via T2A peptides, PCR-generated BsiWI-EcoRI fragment carrying T2A and BsrGI-flanked GFP was cloned into the BsrGI-EcoRI site of pKLV2-EF1dCas9VP64T2ABsd-W and then the BsiWI-BsrGI fragment of pKLV2-MS2p65HSF1T2Ahyg-W, which carries MS2p65HSF1, was inserted into the BsrGI site, resulting in pKVL2-EF1adCas9VP64-t2AMS2p65HSF1-W. In parallel, pENTR-EF1a-GFP_AscIEcoRI-IRESneo was generated as follows: the SalI-HindIII fragment carrying EF1a from pENTR-EF1aCas9-IRESneo [45] and the AscI-NotI-HapI linker were cloned into the SalI-NotI site of pENTR-EF1aCas9-IRESneo, resulting in pENTR-EF1a-ANH-IRESneo, and then the PCR-generated AscI-GFP-EcoRI-NotI fragment was cloned into the AscI-NotI site of pENTR-EF1a-ANH-IRESneo. The AscI-EcoRI fragment carrying dCas9VP64T2AMS2p65HSF1 was cloned into pENTR-EF1a-GFP_AscIEcoRI-IRESneo, resulting pENTR-EF1adCas9VP64_T2A_MS2p65HSF1-IRESneopA. The drug selection marker IREneopA was replaced with PCR-generated IRESbsdpA between EcoRI and SpeI sites, resulting pENTR-EF1adCas9VP64_T2A_MS2p65HSF1-IRESbsdpA. Lastly, the insert in the entry vector, pENTR-EF1adCas9VP64_T2A_MS2p65HSF1-IRESbsdpA, was transferred to pPB-R1R2-EM7NeoPheS [46] by the Gateway cloning, resulting pPB-R1R2_EF1adCas9VP64_T2A_MS2p65HSF1-IRESbsdpA.

Expression plasmid pMCV-EF1a_grow_dCas9-GFP_Blast_pA was a kind gift from Mathias Friedrich (Sanger Institute). The histone acetyltransferase p300 core used in Hilton et al. [22] was synthesized as several gBlock DNA fragments (Integrated DNA Technologies) with homology arms for insertion into lenti dCAS-VP64_Blast (Addgene plasmid #61425). C-terminal insertion (relative to dCas9) of the p300 core domain into the linearized lenti dCAS-VP64_Blast plasmid was constructed with the corresponding gBlock DNA fragments using Gibson assembly and the resulting plasmids digested with XbaI. This enabled insertion of the MS2p65HSF1 construct from plasmid pPB-R1R2_EF1adCas9VP64_T2A_MS2p65HSF1-IRESbsdpA. The resulting dCas9-fusion-T2A-MS2p65HSF1 plasmids were subsequently cloned into a kanamycin-resistant pENTR-EF1a-IRESbsd entry vector using restriction enzyme digest with BsiWI and EcoRI (NEB). VP64 or p300 domains were inserted 5′ of the dCas9 coding sequence in the pENTR-EF1a-IRESbsd entry vector by Gibson assembly. Gateway cloning was performed to transfer the dCas9-fusion-T2A-MS2p65HSF1 constructs into the final ampicillin-resistant expression plasmid pPB-R1R2-IRESbsdpA. VP64 fragments were PCR amplified from lenti dCAS-VP64_Blast. NEBuilder HiFi DNA Assembly Cloning Kit (NEB) was used for all Gibson assembly reactions and conducted according to the manufacturer’s protocol. All plasmids were sequence-verified.

All enzymatic digestions were performed in 50 μL reaction volumes with 5 μg DNA, 5 μL 10× digestion buffer, 1 μL of each restriction enzyme, and incubated at 37 °C for at least 6 h. 5′ dephosphorylation was achieved by incubation with Antarctic Phosphatase (NEB) for 30 min at 37 °C followed by inactivation for 5 min at 80 °C. Digested products were separated by gel electrophoresis on a 1.2% agarose gel and the required fragments purified using Qiagen Gel Purification kit (Qiagen).

PCR reactions for generating VP64 fragments were performed in 25 μL reaction volumes with 12.5 μL 2× Q5 Hotstart Hifi Master Mix (NEB), 1 μL each 10 mM sense and anti-sense primers, 1 μL (1 μg) template DNA, and 9.5 μL nuclease-free water (Ambion). The PCR reactions were performed in a Tetrad 2 Thermal Cycler (Bio-Rad), and cycling conditions were as follows: 30 s at 95 °C for initial denaturation, followed by 25 cycles of 30 s at 95 °C for denaturation, 30 s at 60 °C for annealing, 90 s at 72 °C for extension, and 5 min at 72 °C for the final extension. Purification of PCR products was performed with Qiagen PCR Purification kit (Qiagen). All sequencing primers, PCR primers and gBlock sequences are listed in Additional file 1: Table S2.

### Individual guide RNA design

Guide RNA (gRNA) targeting IL1RN previously published in [22] were used as positive controls for transcriptional activation. For the panel of 12 cell surface receptors, potential guides were identified and ranked using CRISPR-ERA. CRISPR-ERA ranks sequences using an on-target S-score based on distance to the transcriptional start site (TSS), and an off-target E-score based on the number of off-target sites [47]. Eight non-overlapping guides most proximal to the TSS of the longest RefSeq isoform were chosen for each gene. Guides targeting the same gene were cloned as a pool using One Shot TOP10 Chemically Competent *E. coli* (Invitrogen) and propagated in liquid culture. In all other experiments, guides were cloned individually and sequence verified before lentiviral production or transfection into cells.

### Individual gRNA cloning

Expression vector of gRNA with an improved scaffold [48] and MS2-binding hairpin loops was constructed by replacing the MluI-BamHI fragment of pKVL2-U6gRNA5(BbsI)-PGKpuroBFP-W [45] with a gBlock fragment containing the human U6 promoter-driven SAM-gRNA cassette with the BbsI cloning site, resulting pKVL2-U6gRNA_SAM(BbsI)-PGKpuroBFP-W.

Individual guides were synthesized as 24 bp oligomers (Sigma Aldrich and IDT) containing complementary overhangs to those generated by BbsI digestion of the gRNA expression vector. These oligomers were 5′ phosphorylated with T4 PNK (NEB) for 30 min at 37 °C before annealing in 1× T4-ligation buffer (NEB) for 50 min at 95 °C before slowly decreasing the temperature to 25 °C at 0.1 °C/s. Annealed oligos were ligated into the lentiviral gRNA vector by incubating with T4 DNA Ligase (NEB) for 4 h at 16 °C.

### Membrane protein gRNA library design

A non-redundant list of 6213 genes encoding membrane proteins were compiled from five sources using lenient thresholds: a mass-spectrometry derived Cell Surface Atlas [49], a bioinformatic construction of the surfaceome [50], a manually curated list of proteins with experimentally verified cell surface localization kindly provided by Laura Wood (Sanger Institute), the transmembrane protein cDNA collection (Origene), and the Human Protein Atlas (filtered for location: plasma membrane) [51]. TSS predictions were selected from Gencode v19 TSS stratified by strict Fantom5 CAGE clusters, and the two broadest peaks per gene were selected [23]. For genes that were not associated with a CAGE peak, ENSEMBL transcripts annotated as “principal” in the APPRIS database were selected instead. Where no transcripts with this criterion were found, all RefSeq transcripts with NM accession numbers were selected. Promoter region sequences (450–50 bp upstream of each TSS) were obtained from the human assembly hg19 in Ensembl using the BiomaRt package. All 19 nucleotide sequences adjacent to an NGG protospacer adjacent motif (PAM) within these sequences were identified. Guides with < 30% or > 75% GC content, polyT sequences (> 3 Ts), or BbsI restriction sites were discarded, and the resulting guides were ranked according to proximity to the TSS peak. Each guide was mapped using BLAT to all promoter regions targeted and guides with exact matches to promoters other than their intended target were removed, with the exception of those targeting genes with shared promoter regions or gene families with similar promoter sequences. As far as possible, seven guides were selected per transcript/peak. Where a gene had six guides or fewer, rules concerning GC content and polyT stretches were relaxed such that every transcript had at least two guides, with only eight genes having two guides per gene. To ensure a high level of transcription by the U6 promoter, a guanine nucleotide was added to the 5′ end of all guide sequences. Five hundred non-targeting control gRNA sequences were selected from gRNA sequences previously published [24] and were designed to have no binding sites in the human genome (up to two mismatches). All gRNA sequences are listed in Additional file 3.

### Membrane protein gRNA library cloning

Fifty-eight thousand five hundred seventy gRNA sequences were synthesized as a complex pool of 77-mer single-stranded DNA oligos (Twist Biosciences). The sequence of each 77-mer oligo was 5′ GCAGATGGCTCTTTGTCCTAGACATCGAAGACAACACCGN_19_ GTTTTAGTCTTCTCGTCGC where N_19_ represents different 19 bp guides. Double-stranded DNA was amplified from 40 ng of ssDNA oligos using primer pair 77-mer_U1 and 77-mer_L1 (Additional file 1: Table S2). Each reaction contained 1 ng ssDNA, 1.25 μL of each primer at 10 μM, 12.5 μL Q5 2× High Fidelity Hot-start Master Mix (NEB), and nuclease-free water to a final volume of 25 μL. Cycling conditions were as follows: 30 s at 98 °C for enzyme activation, followed by 8 cycles of 10 s at 98 °C for denaturation, 15 s at 63 °C for annealing, 15 s at 72 °C for extension, and a final extension for 2 min at 72 °C.

PCR products were purified using Qiagen Nucleotide Removal kit (Qiagen) and digested with BbsI (NEB) overnight. Digested fragments were separated on a 20% TBE PAGE gel (Invitrogen) at 200 V for 1.5 h and the guide-containing 24 bp fragment excised and purified using the crush-and-soak method in 0.3 M NaCl overnight, followed by ethanol precipitation and resuspension in TE. DNA bands in polyacrylamide gels were visualized by incubating the gel in 0.5 μg/mL ethidium bromide for 10 min followed by ultraviolet light exposure on a transilluminator. Ligation of the membrane protein gRNA library into the pKLV2-U6gRNA_SAM(BbsI)-PGKpuroBFP-W expression vector was performed at a 1:5 insert to vector ratio with T4 DNA Ligase for 2 h at 25 °C and transformed into One Shot TOP10 Chemically Competent *E. coli* (Invitrogen) by heat shock at 42 °C. The total number of colony-forming units was estimated, by plating dilutions of the transformed cells, to be 11× the complexity of the library. Transformants were cultured in a liquid culture and DNA preparation performed using a PureLink HiPure Plasmid Filter Maxiprep Kit (Invitrogen), according to the manufacturer’s instructions. To determine the distribution of gRNA in the plasmid library, 10 ng of the plasmid preparation (~ 1 × 10^9^ copies) were used for Illumina sequencing.

### RNA isolation and q-RT-PCR

Relative mRNA expression levels were quantified by reverse transcription and quantitative PCR (qPCR). Total RNA was isolated from approximately 5 × 10^6^ cells per sample using TRIzol Reagent (Ambion) according to the manufacturer’s protocol. One microgram of total RNA was reverse transcribed using SuperScript III First-Strand Synthesis Kit (Invitrogen), treated with RNase H for 20 min at 37 °C to remove remaining RNA, and the resulting cDNA diluted 30-fold in nuclease-free water. qPCR was performed using Sensimix SYBR Low-Rox Kit (Bioline) with 5 μL of diluted cDNA in a final reaction volume of 15 μL. Samples were prepared in 384-well format with two technical replicates for every RNA sample and cycled on a LightCycler 480 Instrument II. Cycling parameters were as follows: 10 min at 95 °C for polymerase activation, followed by 40 cycles of 15 s at 95 °C for denaturation, 15 s at 55 °C for annealing, and 15 s at 72 °C for extension. A melt-curve analysis (from 25 to 95 °C) was performed at the end of the run to check for the presence of primer-dimers or other unwanted products. Primers annealing to *GAPDH* and *CYPA* have been previously published and were used as housekeeping controls [22, 52]. All other primers were designed using Primer-BLAST, with the exception of IL1RN primers which were previously published in [15]. All qPCR primers used are listed in Additional file 1: Table S2. Threshold cycle (Ct) values were determined by the number of cycles needed to reach an arbitrary fluorescence threshold set just above baseline. Relative mRNA expression was determined using the 2ΔΔCt method where target Ct values were first normalized to *GAPDH* and *CYPA* Ct values. Fold changes in target gene mRNA levels were determined by comparing to mock-transfected experimental controls. All q-RT-PCR primers used are listed in Additional file 1: Table S2.

### Recombinant ectodomain construct design

Members of the adhesion GPCR (aGPCR) family were selected for expression based on the following criteria: they possessed a high-scoring signal peptide prediction by SignalP 4.0, lacked known extracellular cleavage sites other than the GPCR proteolysis site (GPS), and had extracellular domains (ECDs) of less than 2000 amino acids. Where the His-Leu-Thr/Ser cleavage sequence in the GPS domain was conserved, a Thr/Ser to Gly mutation was introduced to prevent self-cleavage [34]. Mammalian expression plasmids encoding the entire predicted extracellular region except the signal peptide were synthesized (GeneArt, Invitrogen) and subcloned into both a “bait” plasmid (pMero-Cd4d3+4-BioLHis - Addgene plasmid 50812) producing a monomeric enzymatically monobiotinylated bait when co-transfected with a plasmid encoding a secreted BirA protein (Addgene plasmid 64395), and a “prey” plasmid (pMero-Cd4d3+4-COMP-blac-FLAGHis) which produces a highly avid pentameric beta-lactamase-tagged protein [3, 53]. Both plasmids contained an exogenous signal peptide that facilitates protein secretion, domains 3 and 4 of rat Cd4 as an antigenic tag, and a polyhistidine-sequence for purification [54, 55]. These tags were used for relative quantification and normalization of proteins, as well as forming oligomers for increased avidity.

The GPI-anchor attachment residue of RTN4R, RTN4RL1, and RTN4RL2 was predicted with PredGPI and the entire extracellular regions, including the endogenous signal peptides, were amplified from full-length cDNA constructs (Origene) by PCR (Additional file 1: Table S2) and cloned into bait and prey expression vectors pTT3-Cd4d3+4-BLH (Addgene plasmid 36153) and pTT3-Cd4d3+4-COMP-blac-FLAGHis (Addgene plasmid 71471).

### Cell lines and culture

Suspension and serum-free adapted HEK293-6E cells were routinely cultured in Freestyle media (Invitrogen) supplemented with 25 μg/mL G418 (Invitrogen) and 0.1% Kolliphor and in Freestyle media supplemented with 50 μg/mL G418 and 1% FBS (Invitrogen) after single-cell cloning. Cells were maintained in suspension in shaking incubators at 125 rpm and passaged every 2–3 days. To select stable dCas9-expressing cell lines for screening, HEK293-6E cells were transfected with pPB-R1R2_EF1adCas9VP64_T2A_MS2p65HSF1-IRESbsdpA or pPB-R1R2_EF1aVP64dCas9VP64_T2A_MS2p65HSF1-IRESbsdpA, along with a hyperactive piggyBac transposase (hyPBase) in a 1:5 ratio of transposase to transposon vector. Selection with Blasticidin S (TOKU-E) at 5 μg/mL was initiated 48 h post transfection. Only cells transduced with pPB-R1R2_EF1aVP64dCas9VP64_T2A_MS2p65HSF1-IRESbsdpA and hyPBase gave rise to stable cell lines and were single-cell sorted into 96-well plates with a BD Influx cell sorter (BD Biosciences). Genomic integration was confirmed by PCR with primers targeting the transgene as well as flanking vector regions. This cell line is henceforth referred to as HEK293-V2M, where V2M stands for dCas9 with VP64 × 2 and MS2p65HSF1. Clonally derived lines were expanded, and the clone with the highest CRISPRa activity as evaluated using the CRISPRa GFP reporter assay was selected. All cell lines used in this project were tested and found negative for mycoplasma contamination (Surrey Diagnostics). For cDNA overexpression experiments, plasmids expressing full-length RTN4R, RTN4RL1, and RTN4RL2 were purchased from Origene and transfected into HEK293-6E cells using PEI.

### CRISPRa GFP reporter assay

To quantitative CRISPRa activity, we developed a cell-based reporter assay: briefly, each construct encodes BFP under a constitutive PGK promoter and GFP driven by a minimal CMV promoter preceded with a tetracycline response element (TRE). The empty reporter encodes a non-targeting guide while the reporter encodes a guide complementary to the TRE, such that when expressed in a cell with an active CRISPRa system, the TetO reporter induces an increase in levels of GFP while the “Empty” reporter does not. To construct the empty control vector, the TRE-minimal promoter from pTRE-tight (Clontech), GFP, and U6gRNA_SAM(BbsI) were cloned into the MluI-BamHI site of pKLV2-U6gRNA5(BbsI)-PGKpuroBFP-W [45], resulting in pKLV2-U6gRNA_SAM(BbsI)-TREGFP-PGKpuroBFP-W. sgTetO (5′-GACGTTCTCTATCACTGATA-3′) was cloned into the BbsI site, resulting in pKLV2-U6gRNASAM(gTetO)-TREGFP-PGKpuroBFP-W.

HEK293-6E cells expressing the dCas9/p300/VP64 variants and MS2p65HSF1 fusion proteins were transduced with lentiviruses carrying either reporter, and GFP/BFP expression was analyzed 72 h post transduction by flow cytometry on a BD LSRFortessa flow cytometer (BD Biosciences) as a measure of activation efficiency.

### Lentiviral production and transduction

HEK293-FT cells used for lentivirus packaging were maintained in DMEM with GlutaMAX supplemented with 10% FBS (Invitrogen) and 1% penicillin-streptomycin and passaged every 2–3 days. For virus production, 5 × 10^6^ cells were seeded in a 10-cm plate at day 0 and transfected with 3 μg of transfer plasmid, 9 μg ViraPower lentivirus packaging vectors (Invitrogen) using 36 μl Lipofectamine LTX and 12 μl PLUS reagent diluted in Opti-MEM I (Invitrogen) transfection media. Cells were incubated for 4 h at 37 °C in transfection media before changing to DMEM with 10% FBS. Viral supernatant was harvested 2 days later, filtered, aliquoted, and stored at − 80 °C. Transduction of other cell lines was performed by incubating with a defined volume of virus overnight at 37 °C. Viral titers were determined by transducing HEK293-6E cells with a serial dilution of viral supernatant and quantifying the percentage of BFP-expressing cells on day 2 post-transduction by flow cytometry. Before performing pooled screens, viruses were titered to achieve a multiplicity of infection (MOI) of 0.3; however, it was found that performing small-scale infections in 96-well plates did not scale up linearly, resulting in a higher level of infection than calculated. Instead, 1 × 10^7^ HEK293-V2M cells were transduced with three different volumes of library virus by overnight incubation at 37 °C. Cells were analyzed 2 days post-transduction by flow cytometry, with BFP as a marker for successful transduction, and the volume of virus which resulted in 25–30% BFP-positive cells was chosen. This process was repeated with each batch of virus produced.

For screening, 4 × 10^7^ HEK293-V2M cells were transduced to achieve between 25 and 30% BFP-positive cells (~ 0.3 MOI corresponds to 200× library coverage). An MOI of 0.3 ensured that the majority of infected cells receive one virus per cell. Transduced cells were sorted on day 2 post-transduction on a MoFlo XDP cell sorter (Beckman Coulter) and BFP-positive cells collected. Between 1.0–1.6 × 10^7^ cells were collected for each transduction (166×–266× library coverage) and maintained in media supplemented with 2 μg/mL puromycin (Gibco) to maintain lentiviral construct expression. To determine the effect of gene activation on cell growth, 6 × 10^7^ cells (1000× library coverage) were sampled on days 7, 10, and 12 post-transduction. To compare the distribution of gRNAs in the transduced library with that in the original plasmid library, as well as between different virus preparations, 6 × 10^7^ cells were sampled on day 7 post-transduction with either virus preparation. Although one might expect to see clusters of interacting cells in the library culture caused by the interaction of upregulated receptors, we did not observe any increase in cell aggregation, possibly due to the shear forces produced from this cell line being grown with constant shaking (125 RPM).

### Comparison of dCas9 activators with p300 and VP64

5 × 10^6^ cells were transduced at < 0.3 MOI, with lentivirus-packaged gRNA pools, with each pool of 8 gRNAs targeting one of 12 surface proteins. This was to mimic screening conditions as closely as possible and to avoid synergistic activation caused by the expression of multiple gRNAs targeting the same gene in one cell. Transduction was carried out by incubating viral supernatants with cells at 37 °C overnight (~ 16 h). The next day, cells were reverse-transfected with respective dCas9-activator expression constructs using Lipofectamine LTX and PLUS Reagent (Invitrogen) in a 96-well format and grown for an additional 48 h before analysis by antibody staining and flow cytometry. Transfection efficiency was estimated to be between 70 and 80% from analysis of cells transfected with the non-activating control expressing dCas9-eGFP (data not shown).

### Flow cytometry and fluorescence activated cell sorting

Hybridoma supernatants were obtained from either the International Blood Group Reference Laboratory (National Health Service, UK) or the Developmental Studies Hybridoma Bank (University of Iowa, USA). Purified antibodies were purchased from Abcam, Merck Millipore, or Biolegend. All antibodies used for flow cytometric analysis, along with their provenance, are listed in Additional file 1: Table S3. For immunofluorescent staining, 100 μL of 1 μg/mL primary antibody was incubated with 5 × 10^5^ cells for 1 h at 4 °C. Cells were then washed 1× in PBS-1% BSA before incubation with 100 μL of 0.1 μg/mL phycoerythrin (PE)-conjugated secondary for 1 h at 4 °C. Finally, cells were washed 1× with PBS-1% BSA before being resuspended in PBS without carrier protein and analyzed by flow cytometry. Resuspending in PBS-1% BSA increased the occurrence of instrument blockage, causing fluctuations in fluorescence intensity during acquisition. Samples were analyzed on a LSRFortessa flow cytometer (BD Biosciences), and the resulting data were analyzed using FlowJo (BD Biosciences).

To detect gain-of-function binding to recombinant protein probes or antibodies, 1 × 10^8^ HEK293-V2M cells were assayed between days 7–10 post-transduction. Cells were washed once in PBS-1% BSA, then incubated with 5 mL normalized recombinant protein or 1 μg/mL primary antibodies for 2 h on ice. Cells were washed again with PBS-1% BSA and then incubated in secondary, PE-conjugated antibodies for 1 h on ice. Cells were washed a final time in PBS-1% BSA before cell sorting. For pre-conjugated bait proteins which had been oligomerized around streptavidin-PE, only a single incubation was performed for 2 h on ice. Labeled cells were resuspended in PBS and sorted using a SH800 cell sorter (Sony Biotechnology). Double positive BFP+PE+ cells were collected and stored at − 20 °C before gDNA extraction and sequencing. We have found that recovering a minimum of 1 × 10^6^ cells from sorting approximately 1 × 10^8^ cells at a 5% threshold is needed for reliably detecting interactions.

### Recombinant protein expression and His-tag purification

All expression plasmids were sequence verified and recombinant proteins produced by transiently transfecting HEK293-6E cells. Plasmids encoding bait proteins were co-transfected with a plasmid encoding secreted BirA in a 9:1 ratio as described [56]. HEK293-6E cells were maintained in Freestyle medium (Invitrogen) supplemented with 25 μg/mL G418 (Invitrogen) and 0.1% Kolliphor. Transfections were left for 5 days, and supernatants were harvested and filtered through a 0.2-μm filter. Supernatants containing prey proteins were used neat or diluted without purification while those containing bait proteins were subjected to His-tag affinity purification. Supernatants containing biotinylated bait proteins were incubated with Ni-NTA agarose beads (Jena Bioscience) overnight at 4 °C with constant rotation. One hundred microliters of beads was used for every 50 mL of supernatant. Polypropylene columns (Qiagen) were equilibrated with 2 mL binding buffer (20 mM sodium phosphate buffer, 0.5 mM NaCl, 40 mM imidazole) before addition of the bead-supernatant mixture. Beads were washed with 5 mL binding buffer and proteins eluted in 500 μL of elution buffer (20 mM sodium phosphate buffer, 0.5 mM NaCl, 400 mM imidazole) by incubating for 30 min at room temperature.

### SDS-PAGE and Coomassie staining

To determine the purity and size of bait proteins, sodium dodecyl sulfate-polyacrylamide gel electrophoresis (SDS-PAGE) was performed under reducing conditions with 10 μL of purified protein, followed by Coomassie staining with InstantBLUE Protein Stain (Novus Biologicals). Proteins were first denatured by boiling for 10 min at 70 °C before gel electrophoresis using NuPAGE 4–12% Bis-Tris Gels (Invitrogen) and MOPS buffer.

### Tetramerization of biotinylated proteins

Bait protein concentrations were normalized to the amount of protein needed to saturate 2 μg of streptavidin conjugated to PE (BioLegend). Streptavidin contains four biotin-binding sites, allowing multiple biotinylated bait proteins to be clustered around a single molecule of streptavidin, thereby increasing the avidity of the oligomerized probe for potential binding partners. The concentration of biotinylated bait needed to saturate a fixed amount of streptavidin-PE was determined by enzyme-linked immunosorbent assay (ELISA).

### Enzyme-linked immunosorbent assay

Serial dilutions of each bait protein were incubated with or without 10 ng of streptavidin-PE overnight at 4 °C. The remaining molecules of free biotinylated bait were captured on streptavidin-coated, flat-bottomed 96-well plates (Nunc) for 45 min at room temperature. Immobilized baits were detected by a primary incubation with monoclonal mouse anti-rat CD4 IgG (OX68), which recognizes a conformation-specific epitope on domains 3 and 4 of CD4 present in the bait, followed by a secondary incubation with an alkaline phosphatase-conjugated anti-mouse IgG (Bethyl Laboratories). All incubations were performed for 1 h at room temperature, and plates were washed 3× in PBS-0.1% Tween 20 and 1× in PBS between additions. One hundred microliters of 1 μg/mL alkaline phosphatase substrate (Sigma) dissolved in diethanolamine buffer (0.5 mM MgCl_2_, 10% diethanolamine, pH 9.2) was added to wells, and substrate hydrolysis after 15 min was quantified by measuring absorbance at 405 nm with a FLUOstar Optima plate reader (BMG Biotech). Absorbance at 405 nm was plotted against dilution factor for each bait protein and the highest concentration at which no free biotinylated bait remained after conjugation with streptavidin-PE was selected.

### Genomic DNA extraction and sequencing

For samples with fewer than 1 × 10^6^ cells, cells were resuspended in nuclease-free water at 8 × 10^5^ cells/mL and lysed for 10 min at 95 °C. Lysates were treated with 2 μg/mL Proteinase K for 50 min at 55 °C followed by 10 min at 95 °C for inactivation. Ten microliters of treated lysate was used as template for each 50 μL PCR reaction. For samples between 1 × 10^6^–2 × 10^6^ cells and 5 × 10^7^–6 × 10^7^ cells, column-based purification of genomic DNA (gDNA) was performed with DNeasy Blood and Tissue kit (Qiagen) and Blood and Cell culture DNA maxi kit (Qiagen), respectively. The DNA concentration in the eluate was quantified with a NanoDrop 1000 spectrophotometer (Thermo Fisher Scientific) and 1–2 μg gDNA was used as template for each 50 μL PCR reaction. Multiple individual PCR reactions (8–36) were performed to achieve sufficient coverage of the library. A 298-bp fragment containing the guide RNA sequence was amplified from gDNA. Illumina adapters and barcodes were added in two successive PCR reactions. Cycling conditions for both reactions were as follows: 30 s at 98 °C for enzyme activation, followed by a number of cycles of 10 s at 98 °C for denaturation, 15 s at 61 °C or 66 °C for primer annealing (first and second reactions respectively), 15 s at 72 °C for extension, and a final extension for 2 min at 72 °C. Depending on the type of input (column-purified gDNA or cell lysate), either 25 cycles or 30 cycles were run for the first PCR reaction, respectively. PCR products from the first reaction were purified using Qiagen PCR purification kit and 1 ng of purified product used as template in the second reaction. The second PCR reaction involved 15 cycles of amplification, after which PCR products were size-selected using solid phase reversible immobilization with Agencourt AmPure XP beads (Beckman Coulter) in a 0.7 *v*/*v* ratio of beads to sample. 5 μL of PCR product was analyzed with gel electrophoresis on a 2% agarose gel to confirm for quantity and size after each reaction. No template controls were performed to monitor possible contamination from other sources. Primers containing Illumina adaptors along with 11 bp barcodes were used to allow for multiplexing of up to 10 samples in a single run. Nineteen base pair sequencing was performed with a custom sequencing primer on a HiSeq 2500 in rapid run mode. All primers used for Illumina library preparation and sequencing are listed in Additional file 1: Table S2.

### CRISPRa screen analysis

Raw sequencing reads were converted from CRAM to FASTQ format using the “fasta” function in SAMTools 1.3 (https://sourceforge.net/projects/samtools/files/samtools/1.3/). The 19 bp reads were then aligned to gRNA sequences using the count function in MAGeCK. MAGeCK is a statistical package built for model-based analysis of CRISPR screens and uses a mean-variance function to estimate a null negative binomial distribution for individual gRNA counts. For testing of gene level enrichment, MAGeCK employs a modified Robust-Rank Aggregation approach to evaluate the likelihood that perturbing a particular gene is having an effect in a pooled CRISPR screen [25]. Counts were normalized by total number of reads to account for differences in sequencing depth. Enrichment testing was performed using the test function in MAGeCK without further normalization and with gRNAs grouped by gene rather than TSS. The sequenced plasmid library was used as the control sample for all tests. Using sequences from unsorted libraries at day 7 or day 12 as the control sample gave similar results. Two genes (*ITGB3* and *MEGF10*) were excluded from all analyses except the pooled antibody screens. This is due to contamination of the sequencing libraries leading to inflated read counts of several guides targeting *MEGF10*, while there was enrichment of guides targeting *ITGB3* in several screens where an anti-α_v_β_3_ antibody was included as a positive control. All genes with a false discovery rate (FDR) below 0.1 were considered candidate receptors and secondary validation performed with individually cloned gRNA or overexpression with full-length cDNA encoding the targeted receptor. All gRNA read count data are provided in Additional file 4.

### Annexin V staining

1 × 10^5^ cells were washed 1× in PBS and 1× in binding buffer for Annexin V staining (Invitrogen), before being resuspended in 100 μL binding buffer. Five microliters Annexin V-FITC (eBioscience) was added to 100 μL cell suspension and incubated at room temperature for 10 min. Cells were then washed 1× with 2 mL of binding buffer and resuspended in 200 μl binding buffer for analysis. Five microliters of propidium iodide was added just before analysis by flow cytometry.

### Beta-lactamase-containing prey protein normalization

Prey proteins were normalized using beta-lactamase activity as a proxy by monitoring the time-resolved appearance of the hydrolysis products of the beta-lactamase colorimetric substrate, nitrocefin, which absorb at 485 nm. Serial dilutions of prey supernatants were made in PBS-1% BSA and 20 μL of each dilution incubated with 60 μL of 125 μg/mL nitrocefin (Calbiochem) at room temperature. Absorbance readings at 485 nm were taken once per minute for 20 min and the dilution which caused complete nitrocefin hydrolysis at 10 mins was selected.

### Avidity-based extracellular interaction screen (AVEXIS)

AVEXIS was performed essentially as described in [3]. Briefly, different dilutions of bait proteins were captured on streptavidin-coated plates for 45 min at room temperature. Plates were washed in PBS-1% Tween 20 and normalized prey proteins were added for 1 h at room temperature. Excess prey protein was removed by washing gently with PBS-1% Tween 20 twice and 60 μL of 125 μg/mL nitrocefin added to detect captured prey proteins. Absorbance readings at 485 nm were taken 1 and 2 h after nitrocefin addition. Either rCd200 and rCd200R or hCD97 and hCD55 were used as positive controls, and PBS-1% BSA added in place of either bait or prey was used as a negative control.

### Other statistical analyses

Student’s *t* test was performed in R.

## Additional files

Additional file 1: **Table S1.** A table listing the gRNAs sequences targeting the promoter regions for the named genes. The gene symbol, accession number of the target transcript and chromosomal location are provided. Table S2. A table detailing the sequences of the synthesized DNA fragments and PCR primers used for plasmid construction and sequencing, primers used for q-RT-PCR, and primers for gRNA library preparation and amplification. Table S3. A table providing the sources, and where appropriate, clone names of the primary monoclonal and conjugated secondary antibodies used in this study. (PDF 553 kb)

Additional file 2: **Figure S1.** CRISPR activation enables rapid and stable upregulation of cell surface proteins. **Figure S2.** A CRISPR activation gRNA library targeting membrane-associated proteins. **Figure S3.** Enrichment of gRNAs targeting known receptors in cells selected using their corresponding ligand. **Figure S4.** ADGRB1 directly interacts with all three members of the RTN4R family. (PDF 489 kb)

Additional file 3: A table detailing all the gRNA sequences present in the CRISPRa library. For each named gene, the gRNA sequence is provided together with the chromosomal location it targets and the distance from the transcriptional start site (TSS). (CSV 4536 kb)

Additional file 4: A spreadsheet containing all the raw gRNA read counts for each of the screens performed in this study. The gRNAs and the gene promoter targeted are listed in the rows, and the experiments in the columns: “plasmid” refers to the lentiviral gRNA library counts prior to transformation; “d7” and “d12-transduced” refer to gRNA counts from cells 7 and 12 days after transduction; “8aB_rep” to the three replicates for the pooled monoclonal antibody screen; and the remaining columns list the protein probes used for selection in the screens. (XLSX 5146 kb)

## Abbreviations

AVEXIS: Avidity-based extracellular interaction screen
BSA: Bovine serum albumin
CRISPR: Clustered regularly interspaced short palindromic repeats
CRISPRa: Clustered regularly interspaced short palindromic repeats activation
dCas9: Deactivated Cas9
ELISA: Enzyme-linked immune sorbent assay
FACS: Fluorescence-activated cell sorting
FDR: False discovery rate
FLAG: “DYKDDDDK” epitope
GPCR: G-protein-coupled receptor
GPI: Glycosylphosphatidylinositol
GPS: GPCR proteolysis site
gRNA: Guide RNA
HAT: Histone acetyltransferase
mAbs: Monoclonal antibodies
PE: Phycoerythrin
SDS-PAGE: Sodium dodecyl sulfate-polyacrylamide gel electrophoresis
TSS: Transcriptional start site
