# Author Correction: Pooled extracellular receptor-ligand interaction screening using CRISPR activation

**DOI:** 10.1186/s13059-022-02797-6

**Published:** 2022-10-21

**Authors:** Zheng-Shan Chong, Shuhei Ohnishi, Kosuke Yusa, Gavin J. Wright

**Affiliations:** 1 Cell Surface Signalling Laboratory, Wellcome Sanger Institute, Cambridge, CB10 1SA UK; 2 Stem Cell Genetics Laboratory, Wellcome Sanger Institute, Cambridge, CB10 1SA UK

**Correction: Genome Biol 19, 205 (2018)**

**https://doi.org/10.1186/s13059-018-1581-3**

Following the publication of the original paper [1], the authors reported a typographical error in the sequence of SAMlibrary-HiSeq_50bp-F1 used for NGS library preparation (as listed in Table S1).

The sequence for Primer 1: SAMlibrary-HiSeq_50bp-F1 has two extra bases inserted (in bold) ACACTCTTTCCCTACACGACGCTCTTCCGATCT**AT**CTTGTGGAAAGGACGAAACA

The correct sequence should be:

ACACTCTTTCCCTACACGACGCTCTTCCGATCTCTTGTGGAAAGGACGAAACA
