# Supplementary material for: Pooled extracellular receptor-ligand interaction screening using CRISPR activation
Source: Genome Biol. 2018 Nov 26;19:205. doi: 10.1186/s13059-018-1581-3 (PMC6258485; doi:10.1186/s13059-018-1581-3)
Supplement: Supplementary file 1 — Table S1. A table listing the gRNAs sequences targeting the promoter regions for the named genes. The gene symbol, accession number of the target transcript and chromosomal location are provided. Table S2. A table detailing the sequences of the synthesized DNA fragments and PCR primers used for plasmid construction and sequencing, primers used for q-RT-PCR, and primers for gRNA library preparation and amplification. Table S3. A table providing the sources, and where appropriate, clone names of the primary monoclonal and conjugated secondary antibodies used in this study. (PDF 553 kb) [file 13059_2018_1581_MOESM1_ESM.pdf]

Supplementary Table 1

ID	Gene symbol	Target transcript	sgRNA sequence	Chromosome	Location
SLC4A1-sg1	SLC4A1	NM_000342	GGGTTTGCGAGCTGCCCCTG	17	42345519
SLC4A1-sg2	SLC4A1	NM_000342	GCCAGTGGGGCGGGCAGATT	17	42345561
SLC4A1-sg3	SLC4A1	NM_000342	AAGAGATAACTCTGTTTACT	17	42345629
SLC4A1-sg4	SLC4A1	NM_000342	ACTCACAGCTGTCCAGATGT	17	42345653
SLC4A1-sg5	SLC4A1	NM_000342	GACTCTTCCTTTGTGGATGA	17	42345743
SLC4A1-sg6	SLC4A1	NM_000342	GTTTGATCGCTCTGTCCTCA	17	42345765
SLC4A1-sg7	SLC4A1	NM_000342	GGGAACTGCTCAGCACTCAC	17	42345964
SLC4A1-sg8	SLC4A1	NM_000342	AGTCTGGATCAAGGAGGGGA	17	42346002
RHD-sg1	RHD	NM_001127691	GCCTGAGATAAGGCCTTTGG	1	25598918
RHD-sg2	RHD	NM_001127691	TCCGTGTTAACTCCATAGAG	1	25598875
RHD-sg3	RHD	NM_001127691	GCACAGCAGGAACCTGTAAC	1	25598775
RHD-sg4	RHD	NM_001127691	GGATTATGTTTGGGTGTCAA	1	25598727
RHD-sg5	RHD	NM_001127691	CATTGTTGTTAAGAGCTCAC	1	25598559
RHD-sg6	RHD	NM_001127691	GCCCTCTCTGTCATGTAGTA	1	25598584
RHD-sg7	RHD	NM_001127691	TGGTTGTGCTGGCCTCTCTA	1	25598890
RHD-sg8	RHD	NM_001127691	ATTTCAACTGTGTAACTATG	1	25598456
P2RX7-sg1	P2RX7	NM_002562	GTTTATCACAGCCACATGTG	12	121570578
P2RX7-sg2	P2RX7	NM_002562	GGTGAGGTCATCTGCCAGCC	12	121570533
P2RX7-sg3	P2RX7	NM_002562	ACCATCTTTGTGTAGGCATC	12	121570496
P2RX7-sg4	P2RX7	NM_002562	GACCAAAAAAGTGAAAGGAA	12	121570433
P2RX7-sg5	P2RX7	NM_002562	CTCATGTCTCTTGGGAGAAA	12	121570414
P2RX7-sg6	P2RX7	NM_002562	AGTCCTTTTCTGAGGCATAA	12	121570355
P2RX7-sg7	P2RX7	NM_002562	AGCGCCAAGTCCTACGGGCC	12	121570554
P2RX7-sg8	P2RX7	NM_002562	GCATCTGGGGGAGGCCAGCT	12	121570511
ENG-sg1	ENG	NM_000118	CCACCCAGTGACAAAGCCCG	9	130617056
ENG-sg2	ENG	NM_000118	AGCCTTGGAGAGGGTGGGAT	9	130617155
ENG-sg3	ENG	NM_000118	GGCCCCCTGAAAGTTCCCCT	9	130617235
ENG-sg4	ENG	NM_000118	GGAACTACTTTAGCCAAGAC	9	130617256
ENG-sg5	ENG	NM_000118	ATGGGATCAGTGAGCTCAGG	9	130617294
ENG-sg6	ENG	NM_000118	AACCAGTGATCTCAACACAT	9	130617340
ENG-sg7	ENG	NM_000118	ATGCCCGACAAGACGTGAAG	9	130617400
ENG-sg8	ENG	NM_000118	GTCAACTGCACTTAGTAGGC	9	130617428
CD2-sg1	CD2	NM_001767	AGGAACTGAAGTGAGACTGG	1	117297049
CD2-sg2	CD2	NM_001767	GAGGCACGTGGTTAAGCTCT	1	117297013
CD2-sg3	CD2	NM_001767	ACTGTAAAAGATGTAAAGAG	1	117296994
CD2-sg4	CD2	NM_001767	GGCAAAGGAGCACATCAGAA	1	117296920
CD2-sg5	CD2	NM_001767	AATTCTCACACAAAAAAATT	1	117296857
CD2-sg6	CD2	NM_001767	ACTCATAAACACATCTGCTT	1	117296899
CD2-sg7	CD2	NM_001767	AGAGGCTAAGTAGATCACTA	1	117296792
CD2-sg8	CD2	NM_001767	AGTATACCTAAGTGGATAAA	1	117296725
VCAM1-sg1	VCAM1	NM_001078	CTTCCAAGACTATAAAATAC	1	101185088
VCAM1-sg2	VCAM1	NM_001078	TCCTCATCTTCGACTCCAAA	1	101185061
VCAM1-sg3	VCAM1	NM_001078	TATCTTTACTGGAAAGATAA	1	101185037
VCAM1-sg4	VCAM1	NM_001078	GAATCCAATGTGGGTTAAGG	1	101184985
VCAM1-sg5	VCAM1	NM_001078	GAAGCTTTCTGAATCCAATG	1	101184995
VCAM1-sg6	VCAM1	NM_001078	TTCTACTCTGGTTTTTGAAC	1	101184929
VCAM1-sg7	VCAM1	NM_001078	TGAAGCTCCTCTCTCTGTCC	1	101185123

VCAM1-sg8	VCAM1	NM_001078	TGAAATTGCTGCCAAAACAA	1	101184660
PROM1-sg1	PROM1	NM_001145847	GACTGAGGCAGATCCCCACG	4	16085635
PROM1-sg2	PROM1	NM_001145847	ATCAGAGTGCGTCCAGGGCT	4	16085676
PROM1-sg3	PROM1	NM_001145847	GCGTTGCAAGAAGGGAGTGC	4	16085790
PROM1-sg4	PROM1	NM_001145847	ATTCTAAGTAAGGGACTCTG	4	16085830
PROM1-sg5	PROM1	NM_001145847	CAGAAGGGTCTAATGCGGCC	4	16085886
PROM1-sg6	PROM1	NM_001145847	GAGGCGCAAGCGTTGCAAGA	4	16085799
PROM1-sg7	PROM1	NM_001145849	GCAAGGCCTCCAGCCTAATC	4	16077832
PROM1-sg8	PROM1	NM_001145849	GCGTGTAAACTGCCTGCACC	4	16077776
SEMA7A-sg1	SEMA7A	NM_001146029	CGCTTGGGTCTGCCTGCGGC	15	74726312
SEMA7A-sg2	SEMA7A	NM_001146029	AGGCGAGAAAAGGCTGCGAG	15	74726380
SEMA7A-sg3	SEMA7A	NM_001146029	AGCGAGAGCGGAACTGCTGG	15	74726414
SEMA7A-sg4	SEMA7A	NM_001146029	GAACCTTCGCCACCCTCTCC	15	74726436
SEMA7A-sg5	SEMA7A	NM_001146029	GCTTTCCCCGTAGAGTTGCC	15	74726458
SEMA7A-sg6	SEMA7A	NM_001146029	AGTCTGGCTTGTCCGCAGCT	15	74726480
SEMA7A-sg7	SEMA7A	NM_001146029	GATTGGCGTAGAAGTCGTGG	15	74726575
SEMA7A-sg8	SEMA7A	NM_001146029	ACCTCTCTCCAAGGGCGCAG	15	74726598
CD200-sg1	CD200	NM_001004196	GACAGCCTCCGCTCCTGTGA	3	112051884
CD200-sg2	CD200	NM_001004196	GAGCGGAGGCTGTCTGTGTG	3	112051878
CD200-sg3	CD200	NM_005944	GAGAAAGGAAATGAGGTGGG	3	112051716
CD200-sg4	CD200	NM_001004196	CACTTTGTCAGTTTCCCCAG	3	112051787
CD200-sg5	CD200	NM_001004196	AGCTCTTGATGTAGTGGCAA	3	112051667
CD200-sg6	CD200	NM_001004196	CAGTCCAGGTAGCAGGAAAA	3	112051735
CD200-sg7	CD200	NM_001004196	ATCCTCATCATTAATGCAAG	3	112051485
CD200-sg8	CD200	NM_001004196	AGAATTGATCACATCATGAA	3	112051526
ICAM1-sg1	ICAM1	NM_000201	ACTTAATAAACCGCTTAGCG	19	10381479
ICAM1-sg2	ICAM1	NM_000201	GAGGCCTGCGTAAGCTGGAG	19	10381346
ICAM1-sg3	ICAM1	NM_000201	ATAACAGTCTCCACTCTCCG	19	10381435
ICAM1-sg4	ICAM1	NM_000201	GTTCGGACCCCCTCGCAGCC	19	10381379
ICAM1-sg5	ICAM1	NM_000201	GCTCATCCACTCGATTAAAG	19	10381327
ICAM1-sg6	ICAM1	NM_000201	GGGAGCCCGGGGAGGATTCC	19	10381285
ICAM1-sg7	ICAM1	NM_000201	ACGTCCACACCTAGCTGACA	19	10381235
ICAM1-sg8	ICAM1	NM_000201	ATCCCTCAGTGGAGGGAGCC	19	10381272
SELE-sg1	SELE	NM_000450	AAGCAATCCCTCCTATAAAA	1	169703243
SELE-sg2	SELE	NM_000450	AATATCCTCCTATTATTCAC	1	169703266
SELE-sg3	SELE	NM_000450	ATTGTCCACATCCAGTAAAG	1	169703283
SELE-sg4	SELE	NM_000450	GAAAGTTTTTGGATGCCATT	1	169703314
SELE-sg5	SELE	NM_000450	GATATTCCCGGGAAAGTTTT	1	169703325
SELE-sg6	SELE	NM_000450	GCATATACGATATAAAGGCA	1	169703410
SELE-sg7	SELE	NM_000450	ATTAGAATTTCAGAAACAGA	1	169703548
SELE-sg8	SELE	NM_000450	GATTTCCTCTTTACTGGATG	1	169703291
KEL-sg1	KEL	NM_000420	CAGCTTCTCAGGGGAGAAGA	7	142659512
KEL-sg2	KEL	NM_000420	GACCAAGGGCAAGATTGCTT	7	142659553
KEL-sg3	KEL	NM_000420	AATACAGAAGAAATGAGAGA	7	142659638
KEL-sg4	KEL	NM_000420	GGGGAGCACCAGACCGACAA	7	142659723
KEL-sg5	KEL	NM_000420	ACACTAAACCTTTGTCGGTC	7	142659734
KEL-sg6	KEL	NM_000420	GAGTCACAGTGCAAGACAAA	7	142659580
KEL-sg7	KEL	NM_000420	TTAGAAATAAAGGAACTTCA	7	142659606
KEL-sg8	KEL	NM_000420	GTCTTTGGCTTTGTTTGCCT	7	142660016

Supplementary Table 2

Name	Sequence (5' - 3')
**p300 core domain gBlock sequences**	
C-terminal insertion 5' fragment 1 (replace VP64)	aagaggaaggtggcgggaggtggaagcggaggaATTTTCAAACCAGAAGAACTACGACAGGCACTGATGCCAAC<br>TTTGGAGGCACTTTACCGTCAGGATCCAGAATCCCTTCCCTTTCGTCAACCTGTGGACCCTCAGCTTTTA<br>GGAATCCCTGATTACTTTGATATTGTGAAGAGCCCCATGGATCTTTCTACCATTAAGAGGAAGTTAGAC<br>ACTGGACAGTATCAGGAGCCCTGGCAGTATGTCGATGATATTTGGCTTATGTTCAATAATGCCTGGTTA<br>TATAACCGGAAAACATCACGGGTATACAAATACTGCTCCAAGCTCTCTGAGGTCTTTGAACAAGAAATT<br>GACCCAGTGATGCAAAGCCTTGGATACTGTTGTGGCAGAAAGTTGGAGTTCTCTCCACAGACACTGTG<br>TTGCTACGGCAAACAGTTGTGCACAATACCTCGTGATGCCACTTATTACAGTTACCAGAACAGGTATCA<br>TTTCTGTGAGAAGTGTTTCAATGAGATCCAAGGGGAGAGCGTTTCTTTGGGGGATGACC
C-terminal insertion 5' fragment 2 (after VP64)	gacctggacatgctgggaggtggaagcggaggaggaggtggaagcggaggaATTTTCAAACCAGAAGAACTACGACAG<br>GCACTGATGCCAACTTTGGAGGCACTTTACCGTCAGGATCCAGAATCCCTTCCCTTTCGTCAACCTGTG<br>GACCCTCAGCTTTTAGGAATCCCTGATTACTTTGATATTGTGAAGAGCCCCATGGATCTTTCTACCATTA<br>AGAGGAAGTTAGACACTGGACAGTATCAGGAGCCCTGGCAGTATGTCGATGATATTTGGCTTATGTTC<br>AATAATGCCTGGTTATATAACCGGAAAACATCACGGGTATACAAATACTGCTCCAAGCTCTCTGAGGT<br>CTTTGAACAAGAAATTGACCCAGTGATGCAAAGCCTTGGATACTGTTGTGGCAGAAAGTTGGAGTTCT<br>CTCCACAGACACTGTGTTGCTACGGCAAACAGTTGTGCACAATACCTCGTGATGCCACTTATTACAGTT<br>ACCAGAACAGGTATCATTTCTGTGAGAAGTGTTTCAATGAGATCCAAGGGGAGAGCGTTTCTTTGGGG<br>GATGACC
C-terminal insertion common 3' fragment	TCTTTGGGGGATGACCCTTCCCAGCCTCAAACTACAATAAATAAAGAACAATTTTCCAAGAGAAAAAAT<br>GACACACTGGATCCTGAACTGTTTGTTGAATGTACAGAGTGCGGAAGAAAGATGCATCAGATCTGTGT<br>CCTTCACCATGAGATCATCTGGCCTGCTGGATTCGTCTGTGATGGCTGTTTAAAGAAAAGTGCACGAA<br>CTAGGAAAGAAAATAAGTTTTCTGCTAAAAGGTTGCCATCTACCAGACTTGGCACCTTTCTTGAGAATC<br>GTGTGAATGACTTTCTGAGGCGACAGAATCACCCTGAGTCAGGAGAGGTCACTGTTAGAGTAGTTCAT<br>GCTTCTGACAAAACCGTGGAAGTAAAACCAGGCATGAAAGCAAGGTTTGTGGACAGTGGAGAGATG<br>GCAGAATCCTTTCCATACCGAACCAAAGCCCTCTTTGCCTTTGAAGAAATTGATGGTGTTGACCTGTGC<br>TTCTTTGGCATGCATGTTCAAGAGTATGGCTCTGACTGCCCTCCACCCAACCAGAGGAGAGTATACATA<br>TCTTACCTCGATAGTGTTCATTTCTTCCGTCCTAAATGCTTGAGGACTGCAGTCTATCATGAAATCCTAA<br>TTGGATATTTAGAATATGTCAAGAAATTAGGTTACACAACAGGGCATATTTGGGCATGTCCACCAAGT<br>GAGGGAGATGATTATATCTTCCATTGCCATCCTCCTGACCAGAAGATACCCAAGCCCAAGCGACTGCA<br>GGAATGGTACAAAAAAATGCTTGACAAGGCTGTATCAGAGCGTATTGTCCATGACTACAAGGATATTT<br>TTAAACAAGCTACTGAAGATAGATTAACAAGTGCAAAGGAATTGCCTTATTTCGAGGGTGATTTCTGG<br>CCCAATGTTCTGGAAGAAAGCATTAAGGAACTGGAACAGGAGGAAGAAGAGAGAAAACGAGAGGAA<br>AACACCAGCAATGAAAGCACAGATGTGACCAAGGGAGACAGCAAAAATGCTAAAAAGAAGAATAAT<br>AAGAAAACCAGCAAAAATAAGAGCAGCCTGAGTAGGGGCAACAAGAAGAAACCCGGGATGCCCAAT<br>GTATCTAACGACCTCTCACAGAAACTATATGCCACCATGGAGAAGCATAAAGAGGTCTTCTTTGTGATC<br>CGCCTCATTGCTGGCCCTGCTGCCAACTCCCTGCCTCCCATTGTTGATCCTGATCCTCTCATCCCCTGCG<br>ATCTGATGGATGGTCGGGATGCGTTTCTCACGCTGGCAAGGGACAAGCACCTGGAGTTCTCTTCACTC<br>CGAAGAGCCCAGTGGTCCACCATGTGCATGCTGGTGGAGCTGCACACGCAGAGCCAGGACGAGGGC<br>AGAGGAAGTCTCCTAACATGCGGTGACGTGGAGGAGAATCCTGGCCCAggaggtatggcttcaaactttactc<br>agtTCTAGAtgtctcctGTACATGAGgaattccgatatcaagcttatcgg

p300 core full	tcgtggGAAGCTTGGggccaccatgATTTTCAAACCAGAAGAACTACGACAGGCACTGATGCCAACTTTGG<br>AGGCACTTTACCGTCAGGATCCAGAATCCCTTCCCTTTCGTCAACCTGTGGACCCTCAGCTTTTAGGAA<br>TCCCTGATTACTTTGATATTGTGAAGAGCCCCATGGATCTTTCTACCATTAAGAGGAAGTTAGACACTG<br>GACAGTATCAGGAGCCCTGGCAGTATGTCGATGATATTTGGCTTATGTTCAATAATGCCTGGTTATATA<br>ACCGGAAAACATCACGGGTATACAAATACTGCTCCAAGCTCTCTGAGGTCTTTGAACAAGAAATTGAC<br>CCAGTGATGCAAAGCCTTGGATACTGTTGTGGCAGAAAGTTGGAGTTCTCTCCACAGACACTGTGTTG<br>CTACGGCAAACAGTTGTGCACAATACCTCGTGATGCCACTTATTACAGTTACCAGAACAGGTATCATTT<br>CTGTGAGAAGTGTTTCAATGAGATCCAAGGGGAGAGCGTTTCTTTGGGGGATGACCCTTCCCAGCCTC<br>AAACTACAATAAATAAAGAACAATTTTCCAAGAGAAAAAATGACACACTGGATCCTGAACTGTTTGTT<br>GAATGTACAGAGTGCGGAAGAAAGATGCATCAGATCTGTGTCCTTCACCATGAGATCATCTGGCCTGC<br>TGGATTCGTCTGTGATGGCTGTTTAAAGAAAAGTGCACGAACTAGGAAAGAAAATAAGTTTTCTGCTA<br>AAAGGTTGCCATCTACCAGACTTGGCACCTTTCTAGAGAATCGTGTGAATGACTTTCTGAGGCGACAG<br>AATCACCCTGAGTCAGGAGAGGTCACTGTTAGAGTAGTTCATGCTTCTGACAAAACCGTGGAAGTAAA<br>ACCAGGCATGAAAGCAAGGTTTGTGGACAGTGGAGAGATGGCAGAATCCTTTCCATACCGAACCAAA<br>GCCCTCTTTGCCTTTGAAGAAATTGATGGTGTTGACCTGTGCTTCTTTGGCATGCATGTTCAAGAGTAT<br>GGCTCTGACTGCCCTCCACCCAACCAGAGGAGAGTATACATATCTTACCTCGATAGTGTTCATTTCTTCC<br>GTCCTAAATGCTTGAGGACTGCAGTCTATCATGAAATCCTAATTGGATATTTAGAATATGTCAAGAAAT<br>TAGGTTACACAACAGGGCATATTTGGGCATGTCCACCAAGTGAGGGAGATGATTATATCTTCCATTGC<br>CATCCTCCTGACCAGAAGATACCCAAGCCCAAGCGACTGCAGGAATGGTACAAAAAAATGCTTGACAA<br>GGCTGTATCAGAGCGTATTGTCCATGACTACAAGGATATTTTTAAACAAGCTACTGAAGATAGATTAA<br>CAAGTGCAAAGGAATTGCCTTATTTCGAGGGTGATTTCTGGCCCAATGTTCTGGAAGAAAGCATTAAG<br>GAACTGGAACAGGAGGAAGAAGAGAGAAAACGAGAGGAAAACACCAGCAATGAAAGCACAGATGT<br>GACCAAGGGAGACAGCAAAAATGCTAAAAAGAAGAATAATAAGAAAACCAGCAAAAATAAGAGCAG<br>CCTGAGTAGGGGCAACAAGAAGAAACCCGGGATGCCCAATGTATCTAACGACCTCTCACAGAAACTAT<br>ATGCCACCATGGAGAAGCATAAAGAGGTCTTCTTTGTGATCCGCCTCATTGCTGGCCCTGCTGCCAACT<br>CCCTGCCTCCCATTGTTGATCCTGATCCTCTCATCCCCTGCGATCTGATGGATGGTCGGGATGCGTTTCT<br>CACGCTGGCAAGGGACAAGCACCTGGAGTTCTCTTCACTCCGAAGAGCCCAGTGGTCCACCATGTGCA<br>TGCTGGTGGAGCTGCACACGCAGAGCCAGGACggaggtggaagcggaggatgtacggccaccatga
**PCR primers for VP64 amplification**	
VP64 Forward	TCGTGGGAAGCTTGGGGCCACCATGGACGCATTGGAC
VP64 Reverse	TCATGGTGGCCGTACATCCAGAACCTCCACCCAGCATGTCCAGGTC
**Sequencing primers for dCas9-activator constructs**	
p300 F1	TGCCTCCCATTGTTGATCCT
p300 F2	TTGTGAAGAGCCCCATGGAT
p300 F3	CCCTTCCCAGCCTCAAACTA
p300 F4	TGCTTCTGACAAAACCGTGG
N term dCas9 F1	TCTCAAGCCTCAGACAGTGG
N term dCas9 R1	TGTACTCGTCGGTGATCACG
C term dCas9 F1	ACTTTGACACCACCATCGAC
C term dCas9 R1	CGTCACCGCATGTTAGGAGA
post T2A F1	AGTCTCCTAACATGCGGTGA
post T2A R1	CACACCGGCCTTATTCCAAG
**Primers for initial amplification of gRNA library**	
77-mer_U1	GCAGATGGCTCTTTGTCCTA
77-mer_L1	GCGACGAGAAGACTAAAAC
**Primers for q-RT-PCR**	
SLC4A1 F	GGGCTCAGATCACCGTAGAC
SLC4A1 R	AGGAGGACAGTACCCTTGGT
RHD F	AGGATCAAAAGGGGCTCGTG
RHD R	TGTTCATGTGGTAGTCTGTGTTG

SELE F	GCCTGCAATGTGGTTGAGTG
SELE R	ATTCATGTAGCCTCGCTCGG
KEL F	CTGATAAGCAGGCTCCACCC
KEL R	CTGGAGTGCTCTCTTGGCTC
**Primers for amplification of RTN4R family ectodomains**	
RTN4R F	CCAAGTTTAAACTGCGGCCGCCACCATGAAGAGGGCGTCCGCTG
RTN4R R	TGGAGGTCGACGGCGCGGGCGCGCCTGAGCCTTCTGAGTCACCAG
RTN4RL1 F	CCAAGTTTAAACTGCGGCCGCCACCATGCTTCGCAAAGGGTGCT
RTN4RL1 R	TGGAGGTCGACGGCGCGGGCGCGCCGCTGGGGGCACG
RTN4RL2 F	CCAAGTTTAAACTGCGGCCGCCACCATGCTGCCCGGGCTCA
RTN4RL2 R	TGGAGGTCGACGGCGCGGGCGCGCCGGAGTCCGGGGGCGCCTGG
**Primers for Illumina library preparation and sequencing**	
First PCR	
SAMlibrary-HiSeq_50bp-F1	ACACTCTTTCCCTACACGACGCTCTTCCGATATATCTTGTGGAAAGGACGAAACA
SAMlibrary-HiSeq_50bp-R1	TCGGCATTCCTGCTGAACCGCTCTTCCGATCTCAGACTGCCTTGGGAAAAG
Second PCR	
HiSeq-PE 1.0	AATGATACGGCGACCACCGAGATCTACACTCTTTCCCTACACGACGCTCTTCCGATC*T
HiSeq-iPCRTag-11mer	CAAGCAGAAGACGGCATACGAGATNNNNNNNNNNNGAGATCGGTCTCGGCATTCCTGCTGAACCGCTCTTCCGATC*T
* indicates phosphorothioate	
Sequencing primer	
U6-SAMlibrary-Illumina-seq (19bp-SE)	TATATCTTGTGGAAAGGACGAAACACCG

Supplementary Table 3

Monoclonal antibodies

Clone	Source	Cat No.	Antigen gene symbol	Antigen gene name
BRAC18	IBGRL	-	SLC4A1	Solute Carrier Family 4 Member 1
BRAD2	IBGRL	-	RHD	Rh blood group D antigen
BRIC18	IBGRL	-	KEL	Kell blood group glycoprotein
P2X7-L4	DSHB	-	P2RX7	P2X purinoceptor 7
P3D1	DSHB	-	ENG	Endoglin
TS2/18.1.1	DSHB	-	CD2	Cluster of differentiation 2
P3C4	DSHB	-	VCAM1	Vascular cell adhesion molecule 1
HB#7/HC7	DSHB	-	PROM1	Prominin 1
MEM-150	Abcam	ab26012	SEMA7A	Semaphorin 7A
OX-104	Bioline	329202	CD200	Cluster of differentiation 200
P2A4	DSHB	-	ICAM1	Intercellular Adhesion Molecule 1
1.2B6	Santa Cruz	sc-18852	SELE	E-selectin
OX68	from hybridoma	-	rCD4 (d3/4)	Rat CD4 (Domains 3 and 4)
LM609	Merck Millipore	MAB1976	$\alpha_v\beta_3$	Integrin $\alpha_v\beta_3$

IBGRL - International Blood Group Reference Laboratory

DSHB - Developmental Studies Hybridoma Bank

Secondary antibodies and other flow cytometry reagents

Antibody description	Source	Cat No.
anti-mouse-PE	Abcam	ab7002
anti-mouse-AP	Sigma Aldrich	A4656
Streptavidin-PE	Biolegend	405204
Annexin V-FITC	eBioscience	BMS306F1
