# Supplementary material for: Pooled extracellular receptor-ligand interaction screening using CRISPR activation
Source: Genome Biol. 2018 Nov 26;19:205. doi: 10.1186/s13059-018-1581-3 (PMC6258485; doi:10.1186/s13059-018-1581-3)
Supplement: Supplementary file 2 — Figure S1. CRISPR activation enables rapid and stable upregulation of cell surface proteins. Figure S2. A CRISPR activation gRNA library targeting membrane-associated proteins. Figure S3. Enrichment of gRNAs targeting known receptors in cells selected using their corresponding ligand. Figure S4. ADGRB1 directly interacts with all three members of the RTN4R family. (PDF 489 kb) [file 13059_2018_1581_MOESM2_ESM.pdf]

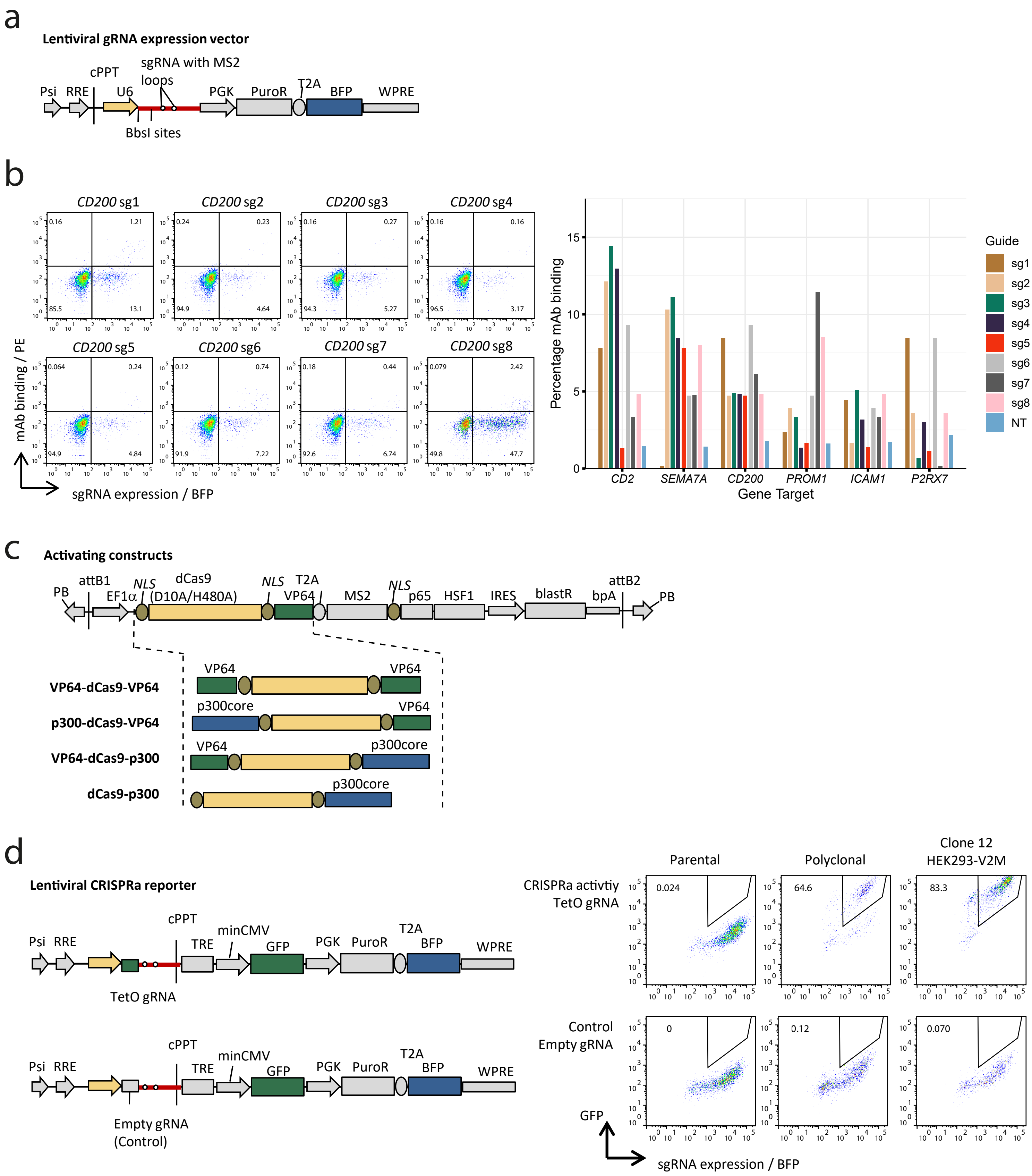

Supplementary Figure 1. CRISPR activation enables rapid and stable upregulation of cell surface proteins. (a) Diagram of the lentiviral gRNA expression vector showing location of the BbsI restriction enzyme sites for cloning the gRNA library, and BFP cell transduction marker. (b) Individual gRNAs exhibit heterogeneity in their ability to upregulate cell surface receptor protein levels by CRISPRa. FACS plots of cells co-transfected with the dCas9-activator plasmid and eight individual single gRNAs (sg) targeting the promoter regions of CD200 (left). Note that individual gRNAs exhibit variability in the number and brightness of cells with upregulated receptors. Quantification of mAb binding to cells co-transfected with dCas9-activator and individual gRNAs for six genes. Bars represent measurements from a single experiment. (c) Schematics showing the single piggyBac-based plasmid construct for expressing dCas9 and MS2 fusion proteins, as well as different dCas9-fusion constructs with combinations of the VP64 and acetyltransferase domain of p300 used in this study. (d) Diagram of the lentiviral gRNA expression vector containing a gRNA (TetO gRNA) that targets the TetO responsive element (TRE) to recruit dCas9 and drive GFP expression as a cellular assay for CRISPRa activity (upper plasmid), an identical virus except containing an "empty" gRNA is used as a control (lower plasmid). Flow cytometry plots of polyclonal or a clonal cell line of HEK cells transfected with the VP64-dCas9-VP64 construct assayed for CRISPRa activity by transducing with the CRISPRa activity GFP reporter lentiviruses "TetO gRNA" or control "Empty gRNA". Note that essentially all BFP+ the cells within the cloned stable cell line (clone 12 - renamed HEK293-V2M) exhibit CRISPRa activity compared to the unselected polyclonal population. (PB, piggyBac inverted terminal repeats; attB1/B2, lambda recombination attachment sites for Gateway cloning; EF1α, Human elongation factor-1 alpha promoter; NLS, Nuclear Localisation Signal; T2A, Self-cleaving peptide; IRES, Internal Ribosomal Entry Site; bpA, Bovine growth hormone polyadenylation site; Psi, Viral packaging signal sequence; RRE, Rev response element; cPPT, Central Polypurine tract; TRE, TetO Responsive Element; PGK, Phosphoglycerate kinase promoter; WPRE, Woodchuck Hepatitis Virus Posttranscriptional Regulatory Element).

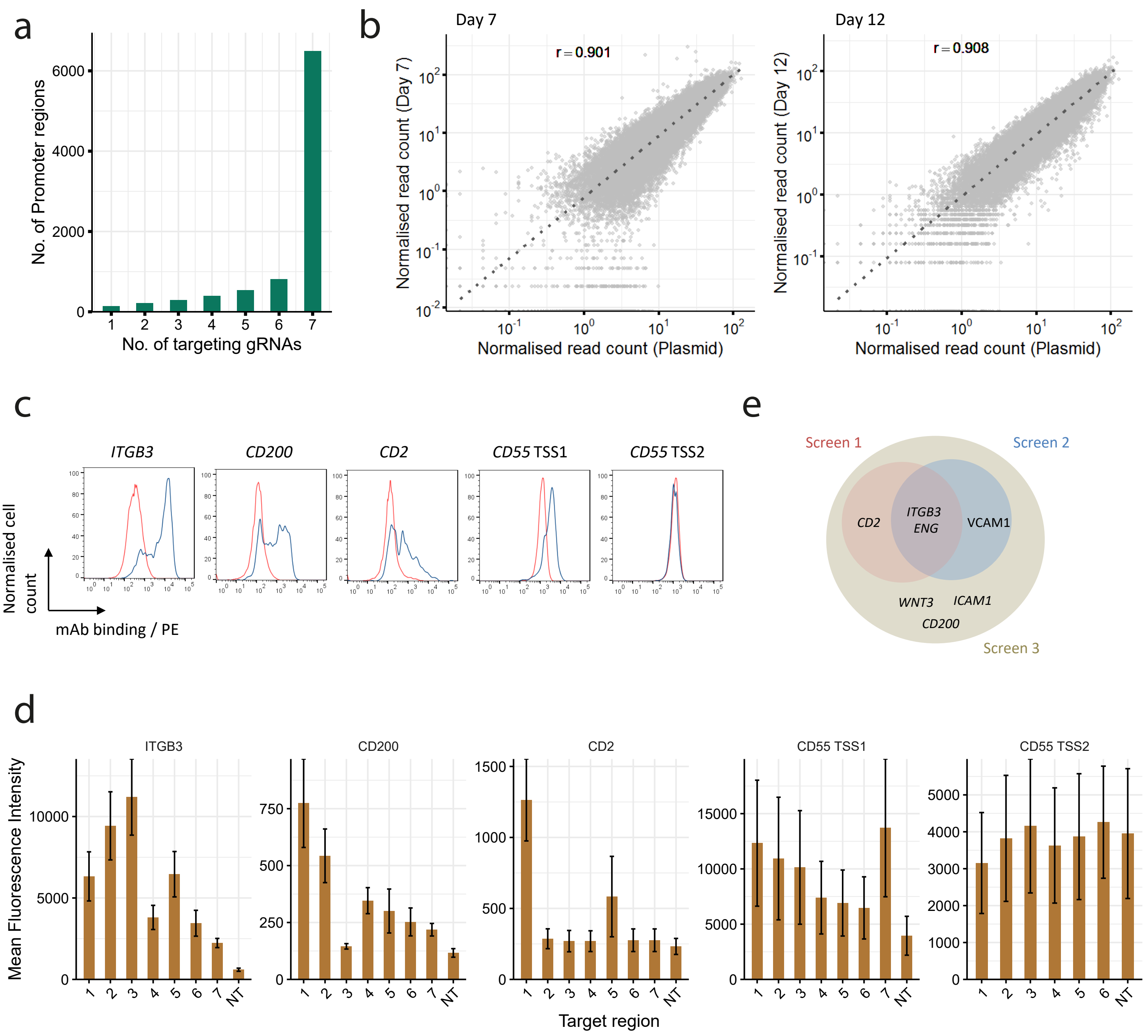

Supplementary Figure 2. A CRISPR activation gRNA library targeting membrane-associated proteins. (a) Number of guides per promoter region of genes predicted to encode cell surface receptor proteins in the membrane protein gRNA library. (b) The gRNA library complexity is maintain in transduced cells. A comparison of the gRNA read count abundance from products amplified from the plasmid library and cells seven and twelve days post transduction. (c) mAb binding histograms of HEK293-V2M cells transfected with the individual gRNAs targeting the promoter region of the named receptor genes (blue traces) compared to control non-targeting gRNAs (red traces) and stained with the respective mAbs. sgRNA number 1 is shown for each target gene. (d) Each gRNA targeting the promoter region of the named receptor proteins were numbered and individually tested and their ability to upregulate cell surface protein expression quantified by FACS compared to a non-targeting (NT) control. (e) Euler diagram summarising the results of three independent genome-wide enrichment screens using a pool of eight mAbs to cell surface receptors. In screens 1 and 2, the most brightly stained 0.5% of cells were selected resulting in the identification of three targets of which ITGB3 and ENG were common to both suggesting undersampling of the library. In screen 3, therefore, the brightest 5% of cells were selected and six out of the eight expected targets were identified as well as one possible false positive, WNT3. The same scoring threshold of FDR ≤ 0.1 was used for all screens.

a

Interaction	Dissociation constant (μM)
CD55 - CD97	86 ± 1[1]
Cd200r - CD200	0.59 ± 0.07[2]
EFNA1 - EPHA2	0.58 ± 0.24[3]
CTLA4 - CD80	0.42 ± 0.06[4]

c

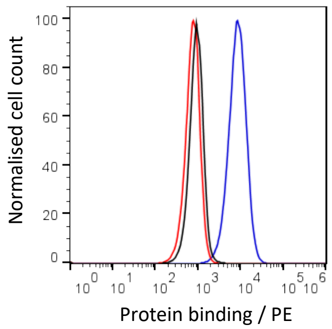

b

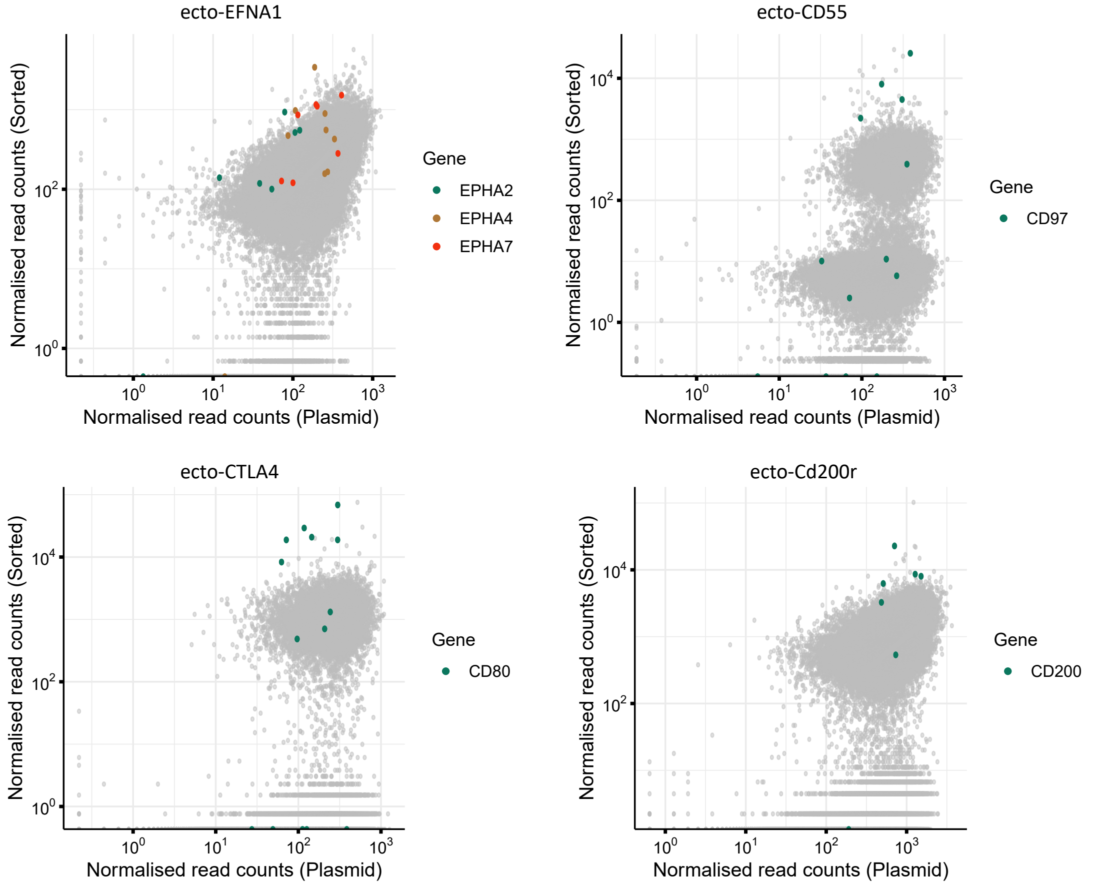

Supplementary Figure 3. Enrichment of gRNAs targeting known receptors in cells selected using their corresponding ligand. (a) Table showing the measured equilibrium dissociation constants and corresponding references for the selected low affinity receptor-ligand interactions that were detected using the genome-wide CRISPRa receptor identification approach. 1Lin HH et al (2001) J Biol Chem. 276(26):24160-9; 2Wright GJ et al (2003) J Immunol. 171(6):3034-46; 3Lema Tomé CM et al (2012) J Biol Chem. 287(17):14012-22; 4van der Merwe PA et al (1997) J Exp Med. 185(3):393-403. (b) Comparison of normalised read counts for gRNAs in amplified PCR products from cells sorted with a highly avid fluorescently-labelled ligand to read counts from the original plasmid library. Ligands were EFNA1 , CD55 , CTLA4 , and Cd200r. (c) HEK293 cells endogenously express an EFNA1 receptor. Recombinant monobiotinylated EFNA1 ectodomain clustered around a streptavidin-phycoerythrin conjugate bound HEK293 cells (blue line). Controls are: unstained cells (black line), streptavidin-phycoerythrin conjugate alone (red line).

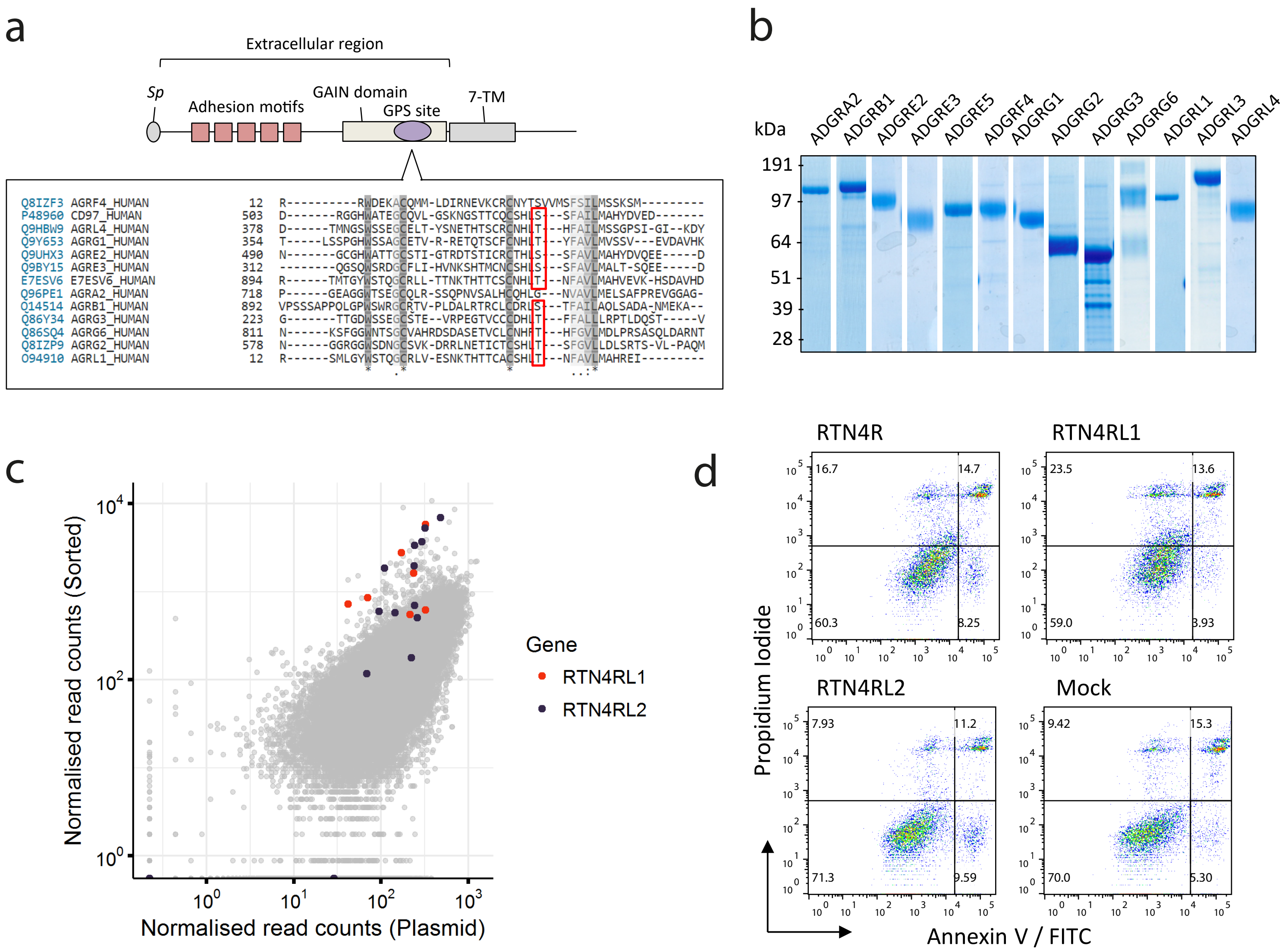

Supplementary Figure 4. ADGRB1 directly interacts with all three members of the RTN4R family. (a) Schematic showing the general architecture of adhesion GPCRs together with a multiple protein sequence alignment of the GPS domain highlighting the location of mutations made to prevent proteolytic cleavage and facilitate recombinant expression of full-length ectodomains. (b) The ectodomains of the named adhesion GPCRs were expressed in HEK293 cells, purified, resolved by SDS-PAGE under reducing conditions and stained with Coomassie blue dye. The major band for each protein corresponded to the expected protein mass. (c) Comparison of normalised read counts for gRNAs in amplified PCR products from cells sorted with the ADGRB1 binding probe to read counts from the original plasmid library. (d) Transfection of cells with cDNAs encoding full-length RTN4R, RTN4RL1, RTN4RL2 did not cause an increase in the levels of cell surface phosphatidylserine, a known ligand of ADGRB1, as determined by Annexin V staining of cells and comparison to mock-transfected cells.
